# CAPN8 involves with exhausted, inflamed, and desert immune microenvironment to influence the metastasis of thyroid cancer

**DOI:** 10.3389/fimmu.2022.1013049

**Published:** 2022-10-27

**Authors:** Xiang Zhong, Shu Xu, Quhui Wang, Long Peng, Feiran Wang, Tianyi He, Changyue Liu, Sujie Ni, Zhixian He

**Affiliations:** ^1^Department of Thyroid and Breast Surgery, Affiliated Hospital of Nantong University, Medical School of Nantong University, Nantong, China; ^2^Department of Oncology, Affiliated Hospital of Nantong University, Medical School of Nantong University, Nantong, China; ^3^Department of Neurosurgery, Affiliated Hospital of Nantong University, Medical School of Nantong University, Nantong, China

**Keywords:** CAPN8, thyroid cancer, prognosis, immunotherapy, tumor immune microenvironment

## Abstract

**Background:**

Thyroid cancer (THCA) is the most prevalent malignant disease of the endocrine system, in which 5-year survival can attain about 95%, but patients with metastasis have a poor prognosis. Very little is known about the role of CAPN8 in the metastasis of THCA. In particular, the effect of CAPN8 on the tumor immune microenvironment (TIME) and immunotherapy response is unclear.

**Material and methods:**

Multiome datasets and multiple cohorts were acquired for analysis. Firstly, the expression and the prognostic value of CAPN8 were explored in public datasets and *in vitro* tumor tissues. Then, hierarchical clustering analysis was performed to identify the immune subtypes of THCA according to the expression of CAPN8 and the activities of related pathways. Subsequent analyses explored the different patterns of TIME, genetic alteration, DNA replication stress, drug sensitivity, and immunotherapy response among the three immune phenotypes. Finally, five individual cohorts of thyroid cancer were utilized to test the robustness and extrapolation of the three immune clusters.

**Results:**

CAPN8 was found to be a significant risk factor for THCA with a markedly elevated level of mRNA and protein in tumor tissues. This potential oncogene could induce the activation of epithelial–mesenchymal transition and E2F-targeted pathways. Three subtypes were identified for THCA, including immune exhausted, inflamed, and immune desert phenotypes. The exhausted type was characterized by a markedly increased expression of inhibitory receptors and infiltration of immune cells but was much more likely to respond to immunotherapy. The immune desert type was resistant to common chemotherapeutics with extensive genomic mutation and copy number variance.

**Conclusion:**

The present study firstly explored the role of CAPN8 in the metastasis of THCA from the aspects of TIME. Three immune subtypes were identified with quite different patterns of prognosis, immunotherapy response, and drug sensitivity, providing novel insights for the treatment of THCA and helping understand the cross-talk between CAPN8 and tumor immune microenvironment.

## Introduction

Thyroid carcinoma (THCA) is the most prevalent malignant disease of the endocrine system, which can be divided into four histological types, including papillary thyroid cancer (PTC), follicular thyroid cancer (FTC), medullary thyroid cancer, and poorly differentiated thyroid cancer (1). The 5-year survival rate for patients with PTC or FTC can attain about 95%, but patients with metastatic THCA have a poor prognosis (2). Calpain calcium kinase (CAPN) is a kind of cysteine protein kinase widely existing in most eukaryotic cells and plays a key role in regulating cell cycle and apoptosis (3). It is already reported that the aberrant expression of CAPN is involved in several types of cancer progression by inducing NF-κB, focal adhesion kinase, and MYC pathways (4–7). However, very little is known about the role of CAPN8 in the genesis and development of THCA up to now. In particular, the effect of CAPN8 on the tumor immune microenvironment (TIME), which is a well-recognized factor in promoting the metastasis of THCA, is unclear (8).

Hereby we hypothesize that CAPN8 might facilitate the metastasis of thyroid cancer cells and lead to poor prognosis by inducing an inhibitory TIME pattern. Firstly, we explored the expression and downstream signaling pathways of CAPN8 in The Cancer Genome Atlas—Thyroid Cancer (TCGA-THCA) cohort and *in vitro* tumor tissues. Next, clustering analysis was performed, and three immune-related clusters (immune exhausted, immune desert, and inflamed) were identified for THCA according to the expression of CAPN8 and related pathways. Subsequent analyses examined the different patterns of genetic alteration, DNA replication stress, TIME, immunotherapy response, drug sensitivity, and prognosis amid the three immune clusters of THCA. Finally, external validation cohorts were utilized to test the robustness and extrapolation of the three immune clusters. Overall, the present study is aimed at elucidating the role of CAPN8 in the metastasis of THCA from the aspects of TIME, DNA replication stress, and genetic variation. These findings will provide novel insights for the treatment of THCA and help understand the cross-talk between CAPN8 and the tumor immune microenvironment.

## Materials and methods

### Multiome dataset acquisition and processing

Multiome datasets of thyroid cancer were obtained from TCGA-THCA (497 tumor samples and 71 normal samples) (9). RNA-seq data, downloaded in the format of fragments per kilobase million at the UCSC Xena website (9), was transformed into the value of transcripts per kilobase million for further analysis. Information about copy number variance (CNV) was acquired from the FireBrowse (10) data portal. Detailed somatic mutation categories were retrieved from the cBioPortal (11) online platform.

Meanwhile, five datasets of thyroid cancer were also exported from the Gene Expression Omnibus database for external validation, including GSE3467 (*n* = 9), GSE3678 (*n* = 7), GSE33630 (*n* = 49), GSE60542 (*n* = 33), and GSE27155 (*n* = 95) cohorts. The batch effect amid different arrays was eliminated by using the ComBat function of R (version 4.1.3) package sva (12). As these data are open access resources from public database where patients’ consents were already obtained, extra informed consents are not needed.

### Immunoreactive score calculation

Firstly, the number of positive cells and the total cells in each stained section were counted to calculate the positive rate (PP) (PP% = positive cells/total cells). By averaging the PP values of 10 discontinuous fields of the experimental tissue in a microscope with a 200-fold high-power lens, the patients were scored with 0 point for no positive cells and 1, 2, 3, and 4 points for 0% < PP ≤ 10%, 11% ≤ PP <50%, 50% ≤ PP < 80%, and PP ≥ 80%, respectively.

Then, the staining intensity (SI) of cells in the tissue was estimated based on the shades of cell color. The SI score was marked as 0 when there was no obvious staining and 1, 2, and 3 for light brownish yellow, brownish yellow, and brown staining, respectively.

The final IRS score was calculated by the following formula: IRS = PP × SI. IRS >3 indicates a high expression, while IRS ≤3 represents a low expression.

### Detecting the mRNA and protein expression of CAPN8 in THCA

The expression difference of CAPN8 between 33 types of cancer and cancer-adjacent tissues was illustrated in a boxplot by using the UCSC Xena web browser. Meanwhile, immunohistochemistry (IHC) was also performed on isolated thyroid cancer tissues to detect the level of CAPN8 protein. IHC was conducted as described previously (13), and tumor sections were obtained from patients who had received radical surgery for thyroid carcinoma in the Affiliated Hospital of Nantong University. The primary anti-bodies used for IHC were anti-CAPN8 (1:30, biorbyt, orb140072).

### Prognostic value and biological function of CAPN8 in THCA

To test the prognostic value of CAPN8, 496 patients in the TCGA-THCA cohort were divided into CAPN8-high and CAPN8-low groups according to the median mRNA value. The Kaplan–Meier (K-m) curve and log-rank test were then utilized to show their difference in progression-free survival (PFS) time.

To explore the biological function of CAPN8, differential expression analysis was carried out between CAPN8-high and CAPN8-low groups by using R package limma (14). |Log_2_ fold change (FC)| >1 and false discovery rate (FDR) <0.05 were set as the significant threshold. Subsequently, Gene Set Enrichment Analysis (GSEA) (15) was performed to recognize the differentially expressed pathways (DEPs) between CAPN8-high and CAPN8-low groups by using R package clusterProfiler. In total, 50 well-known cancer hallmarks (16) were set as the background gene sets, and FDR <0.05 was chosen as the significant threshold.

### Screening for CAPN8-related cancer hallmarks in THCA

To elucidate the potential regulating mechanism of CAPN8 toward thyroid cancer cells, Least Absolute Selection and Shrinkage Operator (LASSO) penalty and ridge regression were implemented to screen the 22 significant DEPs by using R package glmnet (17). In addition, random survival forest (RSF) algorithm was also employed to compute the significance of each DEP by using the R packages randomForestSRC and randomSurvivalForest (https://CRAN.R-project.org/package=beeswarm) with the minimal depth method to determine the final number of prognostic variables. Pathways with a certain contribution to patients’ overall survival were screened in the two models. The importance of each variable was then visualized in a bar plot, and the marginal effect was displayed by the function plot.error of R package randomSurvivalForest.

### Identifying the subtypes of THCA by hierarchical clustering analysis

The six DEPs, obtained by adaptive LASSO regression, and eight DEPs, obtained by random survival forest, were taken into intersection with a Venn diagram depicting the common DEPs by using R package VeenDiagram (18). Gene Set Variation Analysis (GSVA) (15) was then performed to quantify the pathway activities of three common DPEs in THCA. Afterwards, survival analysis was conducted to demonstrate the impact of the three DEPs on PFS. The patients were stratified into two groups according to the median activity score of each DEP, with the K-m curve showing their difference in PFS. Of the common DEPs, HALLMARK_E2F_TARGETS was a prominent risk factor for THCA patients. Therefore, the core enrichment genes of HALLMARK_E2F_TARGETS were then submitted to hierarchical clustering analysis to identify the subtypes of THCA, resulting in an E2F-Clust with two sub-clusters. Hierarchical clustering was completed with Ward’s Clustering, computing the Euclidean distance among each patient by using R function hclust. Consensus Cumulative Distribution Function (CDF) and Delta area (relative change of area under the CDF curve) were used to select the proper clustering numbers. The two indices were provided in R package “ConsensusClusterPlus”.

### Characterizing the different TIME patterns between the two E2F-clusters and three ImmClusters of THCA

GSEA analysis was wielded to dissect the biological features of the two E2F-Clusters of THCA, and survival analysis was conducted to explore their difference in PFS. Moreover, the infiltrating proportions of 10 immune cells were calculated for each TCHA patient by using R package MCPcounter (19) to probe the different TIME pattern (tumor immune microenvironment) between the two E2F-Clusters. In addition, the expression profiles of eight well-known immune inhibitory receptors (IRs)—CD274, PDCD1, CD247, PDCD1LG2, CTLA4, TNFRSF9, TNFRSF4, and TLR9—were also explored in the two E2F-Clusters.

With these immune-related information, the two E2F-Clusters were further subdivided into three clusters (ImmCluster) by using the hclust function in R package ComplexHeatmap (20). Subsequently, the immune enrichment score (IES) and the stromal enrichment score (SES) were compared among the three ImmClusters. IES and SES were obtained by applying ESTIMATE algorithm (21) to each TCHA patient, where IES represented the enrichment score of immune cell ingredients, while SES reflected the ratio of stromal components in tumor tissues. Furthermore, the patients’ diverse responses to immunotherapy were predicted among the three ImmClusters by using Tumor Immune Dysfunction and Exclusion) algorithm (22) and R package Submap (23, 24).

### Resolving the genetic alteration paradigm in two E2F-Clusters and three ImmClusters of THCA

To characterize the different genetic alteration profile among the two E2F-Cluster and three ImmClusters, somatic mutation, CNV, and chromosome instability for each patient were explored by R package MOVICS (Multi-Omics Integration and Visualization in Cancer Subtyping.) (25). CN GISTIC score was also computed for patients in each Immun-Cluster to manifest their different chromosome instability. Specifically, FGA, FGL, and FGG represented the fraction of CN altered genome, fraction of CN-lost genome, and CN-gained genome, respectively.

### Different patterns of DNA replication stress and drug resistance amid two E2F-Clusters and three ImmClusters of THCA

As E2F is part of the cell cycle-related pathways, which can lead to DNA replication stress and drug resistance in cancer cells, we further investigated the activities of 21 pathways related to DNA replication stress (26). Pathway activity was estimated by the GSVA strategy as previously described, and a heat map was utilized to demonstrate the difference between two E2F-Clusters and three ImmClusters. Moreover, the IC50 (half-maximal inhibitory concentration) of five typical chemotherapeutics for THCA was computed and compared between each cluster by using R package pRRophetic (27).

### External validation for the applicability of E2F-Clust and ImmClust in five individual thyroid cancer cohorts

To extrapolate the E2F-Clust and ImmClust for further application in clinical practice, five external datasets of thyroid cancer cohorts were used for validation. One dataset was GSE27155 (*n* = 95) which was quantified by Affymetrix Human Genome U133A Array and annotated by GPL96 platform. The other four datasets—GSE3467 (*n* = 9), GSE3678 (*n* = 7), GSE33630 (*n* = 49), and GSE60542 (*n* = 33) were quantified by Affymetrix Human Genome U133 Plus 2.0 Array and annotated by GPL570 platform. The last four datasets were consolidated by using the ComBat function of R package sva. Principal component analysis was then utilized to visualize the homogeneity of different samples after combination.

Then, based on the core enrichment genes of the E2F pathway, hierarchical clustering analysis was implemented to seek similar subclusters in these datasets. Similarly, patterns of IRs, TIME, DNA replication stress, drug resistance, and immunotherapy responses were explored in different subclusters by using the same analysis strategies as described above.

### Statistic and software

Data processing and bioinformatics analyses were accomplished by R (version 4.1.3). Packages like limma, ggplot2, survminer, clusterProfiler, GSVA, glmnet, MCPcounter, SubMap, MOVICS, *etc.*, were employed for analyses with proper citation. Wilcox or Kruskal–Wallis tests were applied for comparisons between two or more groups involved in this study. Pearson and Spearman rank correlation were adopted to estimate the statistical correlation of parametric or non-parametric variables. A log-rank test was utilized for survival analysis. Two-sided *P <*0.05 was considered the significant threshold for all statistical tests.

## Results

### Expression profile, prognostic value, biological function, and immune features of CAPN8 in THCA

**Figure 1:**
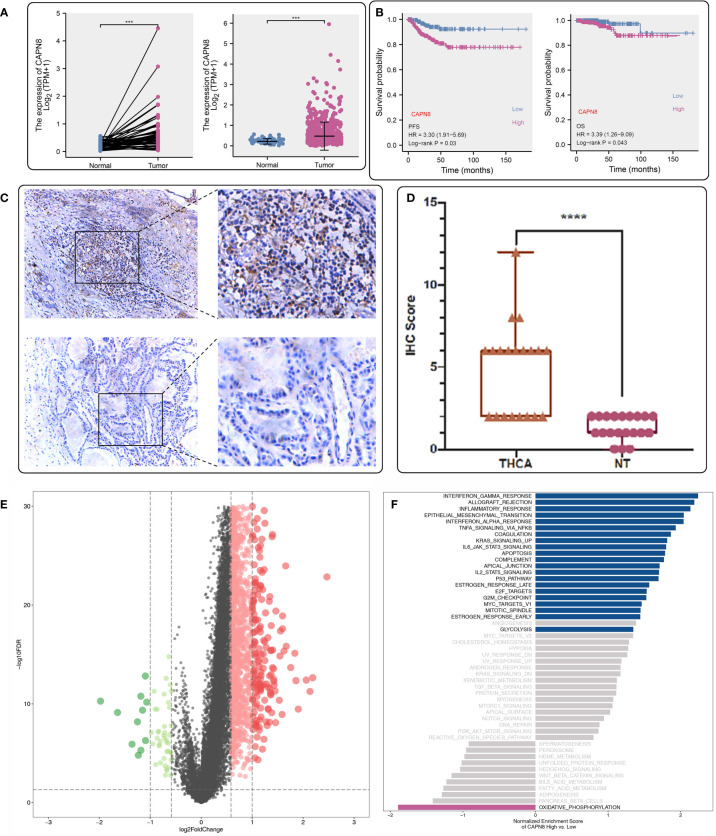
Expression profile and prognostic value of CAPN8 in thyroid cancer. **(A, B, D)** There was an obvious increase in the level of CAPN8 protein **(A)**, mRNA **(B)**, and relative immunohistochemistry score **(D)** in thyroid cancer tissues. **(C)** Influence of CAPN8 expression on the progression-free survival and overall survival of thyroid cancer. **(E)** Differentially expressed genes between the CAPN8-high and CAPN8-low groups. **(F)** Enrichment analysis identified many pathways which were activated in the CAPN8-high group. ***P < 0.001, ****P < 0.0001.

**Figure 2:**
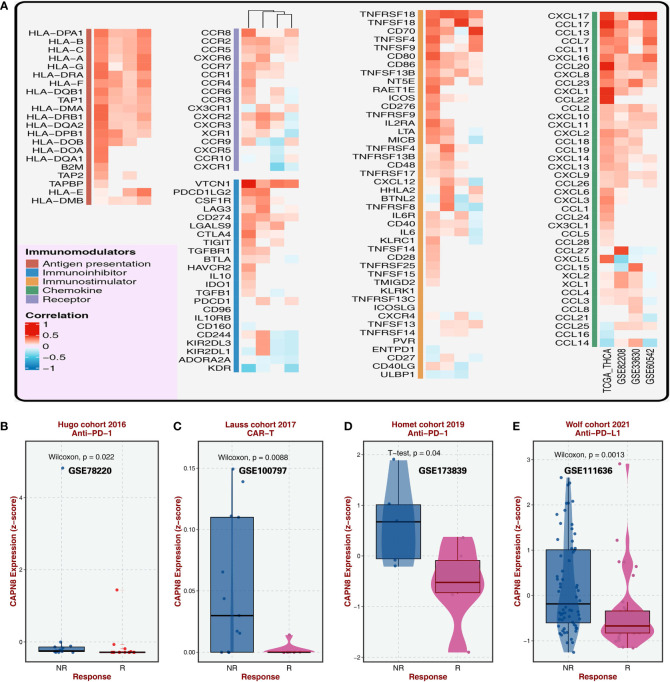
Relationship of CAPN8 with immolators and immunotherapy from the BEST website. **(A)** Relationship of CAPN8 with immunomodulators in the four thyroid cancer cohorts. **(B–E)** Different expression of CAPN8 in different status of patients from four cohorts with immunotherapy.

CAPN8 showed a substantial rise in protein, mRNA, and relative IHC score (D) in thyroid cancer tissues (**Figures 1A, B, D**). Upon dividing the patients into two groups according to the median value of CAPN8 mRNA, patients in the CAPN8-high group were observed to have a noticeable worse survival outcome, suggesting a potential role of oncogene for CAPN8 (**Figure 1C**). The differential expression analysis showed that many genes showed a considerable un-regulation in the CAPN8-high group, while the levels of a relatively few genes decreased (**Figure 1E**). In terms of the 50 cancer hallmarks, interferon-gamma response, inflammatory response, E2F-targets, *etc.*, were significantly upregulated in the CAPN8-high group, while the oxidative phosphorylation pathway was slightly downregulated (**Figure 1F**). We also detected that the strong relationship of CAPN8 with immolators were presented in the BEST website (**Figure 2A**), and CAPN8 could be an immunotherapy predictor for patients who underwent immunotherapy (**Figure 2B**).

### Screening of CAPN8-related signaling pathways and construction of E2F-Clust for THCA

**Figure 3:**
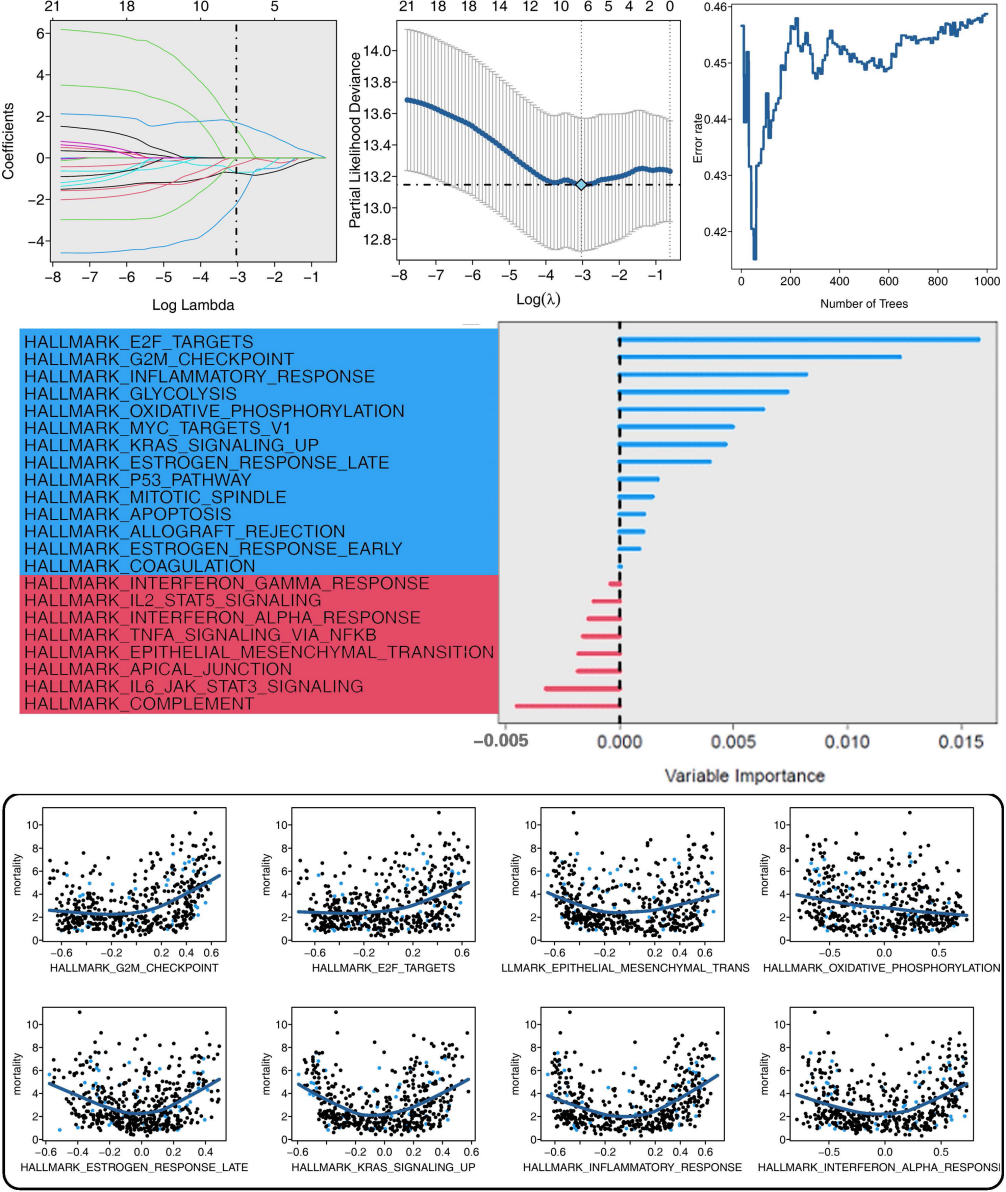
Screening of CAPN8-related hub pathways in thyroid cancer. **(A, B)** Coefficient profiles **(A)** and deviance profiles **(B)** of the adaptive Least Absolute Shrinkage and Selection Operator regression model. Six cancer hallmarks were selected. **(C)** Error rate of the random survival forest (RSF) model. Eight cancer hallmarks were determined. **(D, E)** Importance **(D)** and marginal effect **(E)** of each pathway in the RSF model. The blue color represented pathways with a certain influence on overall survival, while the red color represented background noise with no impact on the dependent variable.

Six and eight CAPN8-related cancer hallmarks were screened out by the adaptive LASSO regression and random survival forest algorithm, respectively (**Figures 3A–C**). Each pathway was graded in order of their importance, and the E2F-targets pathway showed a dominant impact on survival time in the RSF analysis (**Figure 3D**). In addition, the marginal effect of RSF was demonstrated in the scatter diagram where the E2F-target and G2M-checkpoint pathways exhibited a mild positive correlation with the mortality of THCA patients (**Figure 3E**). These findings suggest that the E2F-targets pathway could be the downstream signaling pathway of CAPN8 and plays a key role in THCA progression.

**Figure 4:**
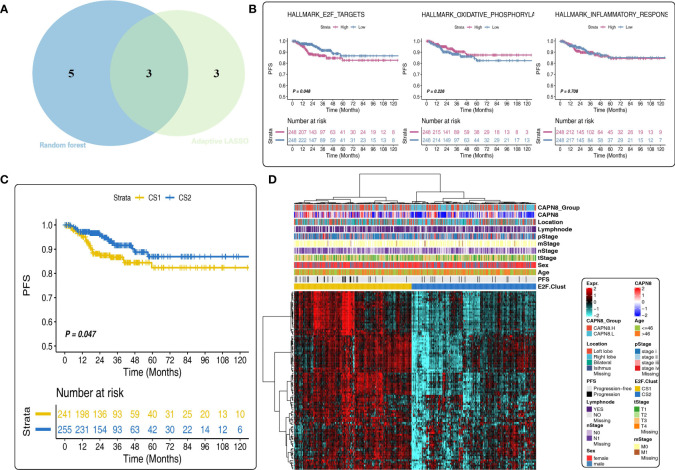
Prognostic value of the E2F-Clust and E2F-targets pathway. **(A)** Three mutual pathways were identified by taking an intersection of the results from the Least Absolute Shrinkage and Selection Operator model and the random survival forest model. **(B)** Prognostic value of three hub pathways. Only E2F-targets pathway was a significant risk factor for thyroid cancer. **(C, D)** E2F-Clust stratified the patients into two groups with a distinct difference in survival outcome **(C)** and clinical TNM stages **(D)**.

Subsequently, three shared cancer hallmarks were documented after taking the intersection of six pathways from LASSO regression and eight pathways from RSF analysis, including E2F-targets, oxidative phosphorylation, and inflammatory-response pathways (**Figure 4A**). Of the three pathways, E2F-targets was a risk factor, and patients with a high pathway activity of E2F-targets demonstrated a worse survival outcome (**Figure 4B**).

A further cluster analysis identified two subtypes of THCA based on the core genes of the E2F-targets pathway (**Figure 4C**). Of the E2F-Clust, CS1 (cluster 1) was characterized by an evidently high expression of CAPN8 as well as core genes in E2F-targets pathways, showing an unfavorable effect on patients’ survival outcome (**Figure 4D**) compared to the superior influence of CS2 (cluster 2). This E2F-Cluster further supported that CAPN8 may lead to THCA progression by regulating the E2F-targets pathway.

### Immune exhausted, immune desert, and inflamed TIME patterns in the two E2F-Clusters and three ImmClusters of THCA

**Figure 5:**
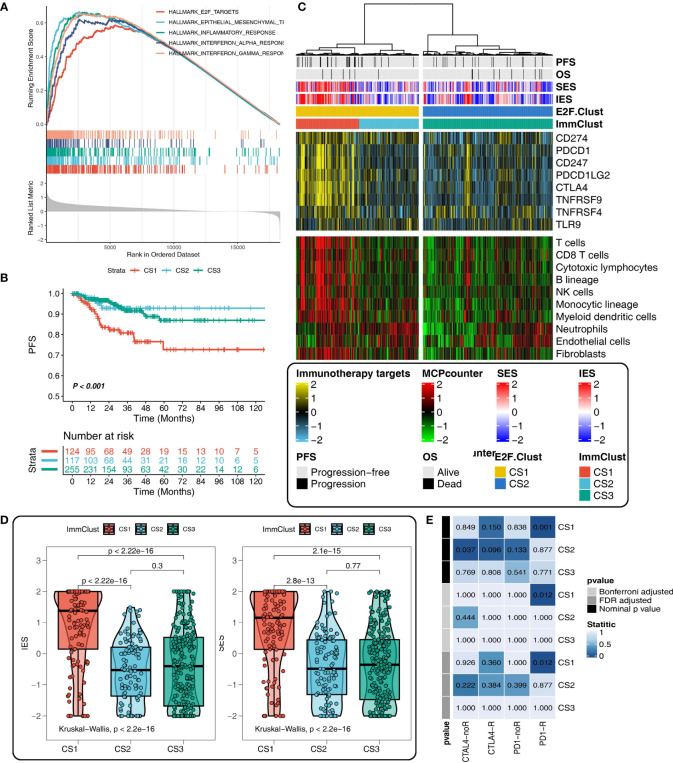
Different tumor immune microenvironment (TIME) patterns in the E2F-Clust and ImmClust. **(A)** The enrichment analysis identified five upregulated pathways in CS1 of E2F-Clust. **(B)** There was a significant difference in PFS among the three ImmClusters. **(C)** Different TIME patterns among the three ImmClusters, including the immune exhausted (CS1), inflammatory (CS2), and immune desert (CS3) subtypes. **(D)** The Immune Enrichment Score and the Stromal Enrichment Score differ in the three ImmClusters. **(E)** Different responses to immunotherapy of the three ImmClusters. OS, overall survival; PFS, progression-free survival; FDR, false discovery rate; TIME, tumor immune microenvironment.

There was a considerable diversity of biological function and TIME pattern between the two E2F-Clusters. CS1 was identified by the elevated pathway activity of E2F-targets, epithelial–mesenchymal transition (EMT), inflammatory response, and interferon-gamma response (**Figure 5A**).

Compared to the two E2F-Clusters, three subtypes were identified in the ImmClust with a significant difference in survival outcome (**Figure 5B**). Considering the information of 10 immune cells and eight IRs, CS1 of the ImmuClust was accompanied with an increased expression of IRs and an infiltrating ratio of almost all types of immune cells, underlying a strongly inhibitory TIME pattern in the CS1 group. This result suggested that CAPN8 could induce T cell exhaustion to inhibit immune response and lead to a poor prognosis of THCA (**Figure 5C**). Moreover, CS1 was characterized by a higher infiltrating proportion of fibroblast than CS2, accounting for its distinctly higher enriched score of SES (**Figure 5D**).

CS2 of the ImmClust, however, was characterized by an inflammatory TIME pattern with high levels of infiltration of neutrophil and endothelial cells. Distinctively, CS3 of the ImmClust lacked immune infiltration, suggesting a potential phenotype of immune desert for this subtype (**Figure 5C**). Keeping consistent with the exhausted TIME feature of CS1, immunotherapy seemed to be a feasible strategy for this subtype. Patients in CS1 significantly responded to PD1-R treatment (**Figure 5E**).

### Genetic alteration paradigm in the two E2F-Clusters and three ImmClusters of THCA

**Figure 6:**
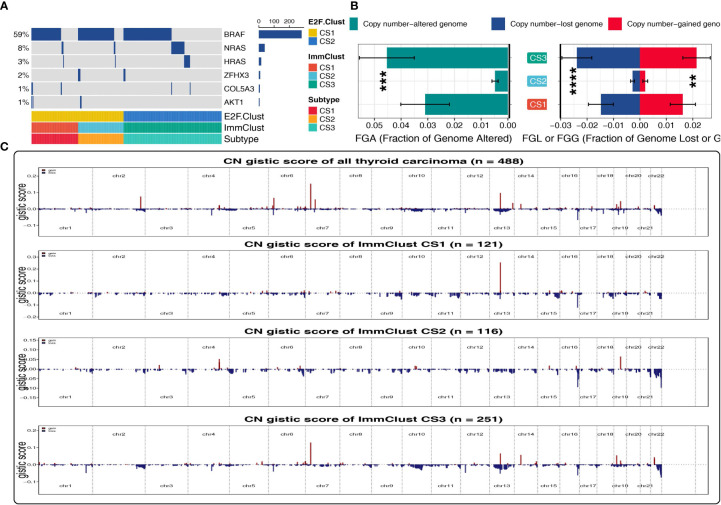
Patterns of genetic alteration in the E2F-Clust and ImmClust. **(A)** Oncoplot showing the mutation information of the E2F-Clust and ImmClust. **(B)** The fraction of copy number variance among the three ImmClusters. **, ***, and **** represent *p* < 0.01, *p* < 0.001, and *p* < 0.0001, respectively. **(C)** Copy number GISTIC score of the E2F-Clust and ImmClust.

Oncoplot was illustrated to resolve the mutation profile of E2F-Clusters and ImmClusters. CS2 of the ImmClust seems to have the highest mutation frequency of BRAF and ZFHX3, while CS1 exceeded the other two clusters in the mutation frequency of COL5A3 and AKT1, which is a hub element of the PI3K proliferation pathway (**Figure 6A**).

By contrast to the mutation frequency, CS2 of the ImmClust was fairly stable in CNV with the lowest FGA, FGL, FGG, and CN GISTIC score than CS1 and CS3 (**Figure 6B**), suggesting a rather recent cancer origin and fairly low chromosomal instability for this subtype.

CS3, however, was in the lead in CNV frequency and CN GISTIC score, underlying a quite earlier cancer origin and extremely high chromosomal instability (**Figure 6C**) for this subtype.

### Patterns of DNA replication stress and drug resistance in two E2F-Clusters and three ImmClusters of THCA

**Figure 7:**
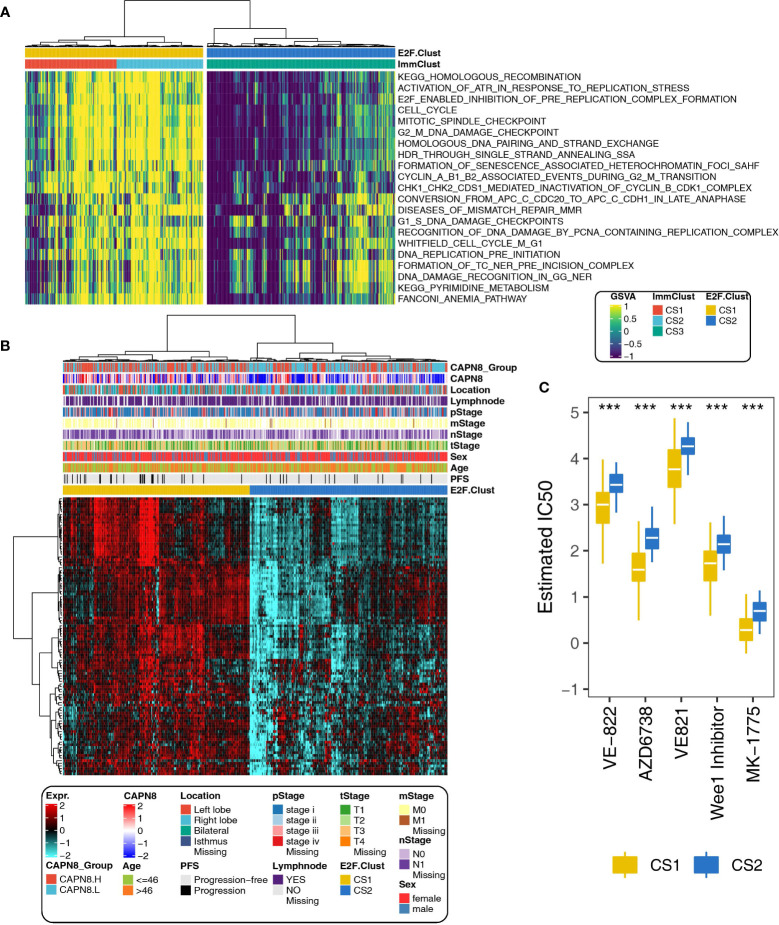
Different paradigms of DNA replication stress and drug sensitivity in the E2F-Clust and ImmClust. **(A)** Activities of 21 pathways related to DNA replication stress in the E2F-Clust and ImmClust. There was evident downregulation of DNA replication stress in the CS3 subtype. **(B)** The E2F-Clust stratified patients into two groups with distinct differences in clinical TNM stages and progression-free survival. **(C)** IC50 (half maximal inhibitory concentration) of typical inhibitors targeting ATR and week 1 protein. Patients of CS2 showed a tendency to be drug resistant. ***P < 0.001.

With regards to the 21 pathways related to DNA replication stress, CS3, the immune desert subtype, was dramatically downregulated in the pathway activity of cell cycle, G1S-DNA damage checkpoints, G2M-DNA damage checkpoint, and mitotic spindle checkpoint, implying a considerably declined ability to maintain the correct paradigm of DNA replication (**Figure 7A**).

This is in line with the result mentioned above, namely: there was a highly altered genomic CNV situation and increased chromosomal instability for CS3 cluster (**Figure 6C**), suggesting that CS3, the immune desert subtype, was prone to being drug resistant by inducing extensive epigenetic variations.

As expected, CS2 of the E2F-Clust, similar to CS3 of the ImmClust, was relatively insensitive to many chemotherapeutics and correlated with poor survival outcome (**Figure 7B**). Specifically, the estimated IC50 values of VE-822, AZD67738, VE821, and MK-1775 were widely increased (**Figure 7C**). These drugs are famous inhibitors of ATR and week1, which are famous cell cycle regulatory proteins.

### Application of the E2F-Clust and ImmClust in five individual thyroid cancer cohorts

**Figure 8:**
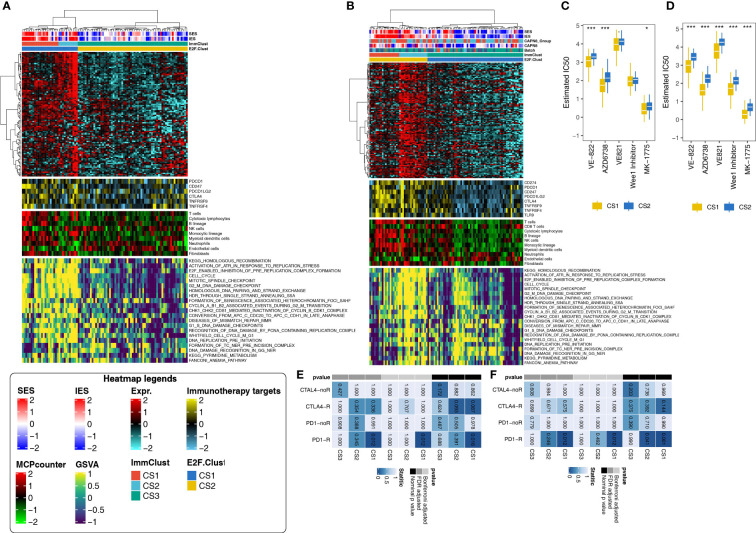
Application of the E2F-Clust and ImmClust in two external validation datasets (*N* = 78, *N* = 95). **(A)** The exhausted, inflamed, and desert TIME patterns remain recognizable after the application of E2F-Clust and ImmClust in the combined validation dataset (*N* = 78). **(B)** The exhausted, inflamed and desert TIME patterns are still distinct after applying the E2F-Clust and ImmClust in GSE27155 (*N* = 95). **(C)** CS2 still showed a tendency to be drug resistant in the combined validation dataset (*N* = 78). **(D)** Patients of CS2 are potentially resistant to common chemotherapeutics in GSE27155 (*N* = 95). **(E)** CS1 is prone to response to anti-PD-1 treatment in the combined validation dataset (*N* = 78). **(F)** Anti-PD-1 treatment is feasible in patients of CS1 subtype in GSE27155 (*N* = 95). *P < 0.05, ***P < 0.001.

The z-score normalization for GSE3467 (*n* = 9), GSE3678 (*n* = 7), GSE33630 (*n* = 49), and GSE60542 (*n* = 33) was appropriate as the heterogeneity among the four datasets was eliminated after combination. After applying the E2F-Clust and ImmClust to this combined thyroid cancer cohort (*n* = 78), the same TIME pattern in the TCGA-THCA cohort was still recognizable. CS3 remained to be the phenotype of immune desert with decreased infiltration of immune cells and expression of IRs. CS1 remained as the phenotype of immune exhausted for its increased infiltration of immune cells and IRs expression, while CS2 maintained the phenotype of inflamed TIME with elevated infiltration of neutrophils (**Figure 8A**).

The pattern of DNA replication stress, however, seems to be indistinguishable in the combined cohort. CS1, CS2, and CS3 were all seemingly accompanied by the increased activity of 21 DNA replication-related pathways (**Figure 8A**). Despite this uncertain result, CS2 was still much more likely to be drug resistant, with generally rising IC50 for ATR and week1 inhibitors (**Figure 8C**). Moreover, immunotherapy was plausible for the CS1 subtype in this combined cohort as patients in the CS1 subtype significantly responded to PD1-R treatment (**Figure 8E**).

Similar patterns of TIME, drug sensitivity, and immunotherapy response were still distinguishable in another validation cohort: GSE27155 cohort (*n* = 95), annotated by GPL96 platform. The immune exhausted inflammatory and immune desert phenotypes still corresponded to CS1, CS2, and CS3 subtypes, respectively (**Figure 8B**). The activities of 21 DNA replication stress-related pathways and drug sensitivity kept decreasing in the CS3 subtype (**Figure 8D**). The CS1 group demonstrated the same certain probability of benefiting from PD1-R treatment (**Figure 8F**).

## Discussion

The present study identified three immune subtypes of THCA according to the expression of CAPN8 and related pathways, including the immune exhausted (CS1), inflamed (CS2), and immune desert (CS3) phenotypes. Three sub-clusters for THCA demonstrated quite diverse patterns of TIME, genetic variation, drug sensitivity, immunotherapy response, and patient prognosis—for instance, patients with CS1, with high expression of CAPN8, demonstrated rather detrimental survival outcomes when receiving chemotherapy but were much more likely to respond to anti-PD-1 treatment. These findings will provide novel insights for the treatment of THCA and help to understand the cross-talk between CAPN8 and the tumor immune microenvironment.

Firstly, CAPN8 was found to be a significant risk factor for THCA with a markedly elevated level of mRNA and protein in tumor tissues. This potential oncogene also induced the activation of EMT and E2F-targets, which are well-acknowledged pathways to promote cancer metastasis and proliferation. Consistent with our studies, CAPN8 was claimed to be a potential oncogene in gastric cancer, hepatic carcinoma, and lung cancer, causing the occurrence of precancerous lesions and cancer progression (5, 6, 28). In addition, members of CAPN family have been reported to facilitate the invasion of THCA by inducing MMP2 and MMP9 secretion, which can contribute to extracellular matrix degradation during cancer cell migration (29).

Furthermore, three immune phenotypes of THCA were identified according to the expression of CAPN8 and related pathways. CS1, the immune exhausted subtype, was accompanied with a distinctly increased expression of IRs and proportion of infiltrating T cells. Accordingly, exhausted T cells lose their killing ability because of the increased expression of IRs but can be restored by immune checkpoint inhibitors (30). This exactly accords with our result: CS1 was found to be positively responsive to anti-PD-1 treatment.

In terms of CS3, this immune desert subtype of THCA demonstrated the absence of anti-tumor immunity and surging level of CNV. This is not surprising as there was a general downregulation of 21 pathways related to DNA replication stress in CS3. It is widely accepted that replication stress plays a key role in initiating anti-tumor immunity by inducing cancer-related neoantigens (31, 32), and its absence is intensively correlated with the immune desert phenotype. Consequently, the low immunity and high level of CNV can jointly contribute to strong cancer stemness (33), accounting for the result that patients of CS3 were tolerant of many ATR and week1 inhibitors in our study.

Overall, three immune subtypes of THCA were identified based on the expression of CAPN8 and related pathways in our study. The three types displayed rather different paradigms of TIME, immune therapy response, drug sensitivity, and genomic variance. Moreover, these three immune subtypes are highly coincident with the results of previous studies on cancer classification (34–38), where the exhausted, inflamed, and desert phenotypes of breast cancer, prostate cancer, and bladder cancer were characterized by using a similar clustering analysis.

Our study has several advantages. This is the first study to elucidate the role of CAPN8 in the metastasis of THCA from the aspects of TIME, DNA replication stress, and genetic alteration. Three immune subtypes identified in our study will provide new insights for the treatment of THCA, as different subtypes showed distinctly different responses to immunotherapy and chemotherapy. Most importantly, external validation in five individual cohorts made the extrapolation and robustness of the classification convincing.

There were also some limitations to the current study. Firstly, further *in vitro* experiments will make it more authentic for the existence of the three subtypes of THCA. Secondly, the prognostic effects of CAPN8 could be validated in actual cohorts of THCA to make it more persuasive. Lastly, analysis on cancer stemness can be added to further explain the relationship between different subtypes and various drug sensitivities.

In conclusion, we highlighted the role of CAPN8 in THCA metastasis and identified three distinct immune subtypes that can be distinguished in terms of prognosis, immunotherapeutic response, and drug sensitivity, which provide new insights for the treatment of THCA and contribute to the understanding of the interaction between CAPN8 and the tumor immune microenvironment.

## Data availability statement

The original contributions presented in the study are included in the article/**Supplementary Materials**. Further inquiries can be directed to the corresponding authors.

## Author contributions

All authors contributed to the study’s conception and design. XZ, SX, and QW performed data collection and analysis. XZ and SX wrote the manuscript. LP, FW, and TH polished and revised the manuscript. ZH and SN provided analytical ideas. All authors contributed to the article and approved the submitted version.

## Funding

This work was supported by grants from the Research Project of Maternal and Child Health of Jiangsu Province (F201953) and the Science and Technology Project of Nantong (JC2020067) to ZH.

## Conflict of interest

The authors declare that the research was conducted in the absence of any commercial or financial relationships that could be construed as a potential conflict of interest.

## Publisher’s note

All claims expressed in this article are solely those of the authors and do not necessarily represent those of their affiliated organizations, or those of the publisher, the editors and the reviewers. Any product that may be evaluated in this article, or claim that may be made by its manufacturer, is not guaranteed or endorsed by the publisher.
