# Supplementary figures and images for: CAPN8 involves with exhausted, inflamed, and desert immune microenvironment to influence the metastasis of thyroid cancer
Source: Front Immunol. 2022 Oct 27;13:1013049. doi: 10.3389/fimmu.2022.1013049 (PMC9647051; doi:10.3389/fimmu.2022.1013049)

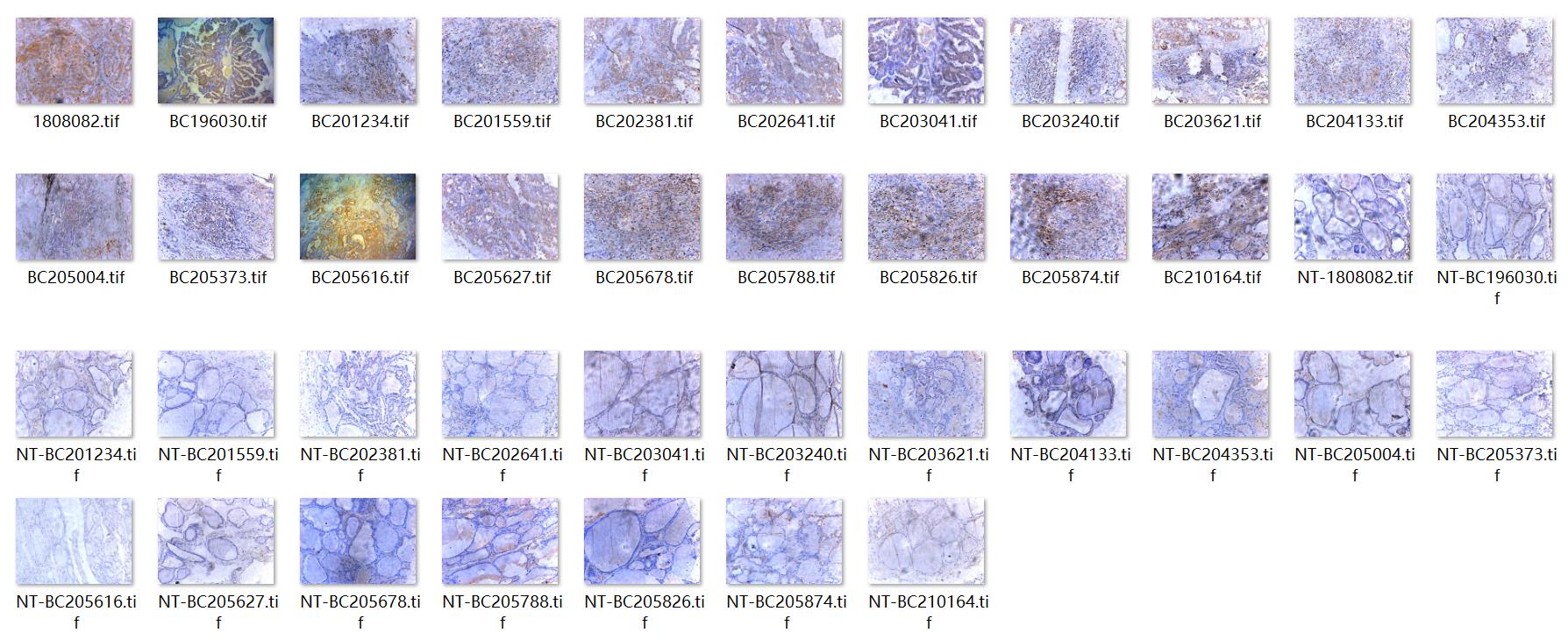

Supplement: Supplementary file 1 [file Image_1.jpeg]
