# Supplementary material for: CAPN8 involves with exhausted, inflamed, and desert immune microenvironment to influence the metastasis of thyroid cancer
Source: Front Immunol. 2022 Oct 27;13:1013049. doi: 10.3389/fimmu.2022.1013049 (PMC9647051; doi:10.3389/fimmu.2022.1013049)
Supplement: Supplementary file 4 [file Table_3.docx]

#-----------------------------#

# ANALYSIS PROJECT: PTC CAPN8 #

# START DATE: 2022/05/01 #

# set working path and related directories

workdir <- "E:/IGBMC/externalproject/ExternalProject/Giotto/PTC_CAPN8"; setwd(workdir)

fig.path <- file.path(workdir,"Figures")

res.path <- file.path(workdir,"Results")

data.path <- file.path(workdir,"InputData")

comAnn.path <- file.path(workdir,"Annotation")

comRFun.path <- file.path(workdir,"commonFun")

# create directories

if (!file.exists(res.path)) { dir.create(res.path) }

if (!file.exists(fig.path)) { dir.create(fig.path) }

if (!file.exists(data.path)) { dir.create(data.path) }

if (!file.exists(comAnn.path)) { dir.create(comAnn.path) }

if (!file.exists(comRFun.path)) { dir.create(comRFun.path) }

# load R packages

library(ComplexHeatmap)

library(ClassDiscovery)

library(survival)

library(survminer)

library(gplots)

library(ggplot2)

library(RColorBrewer)

library(maftools)

library(MOVICS)

library(sva)

library(RTN)

library(CMScaller)

library(limma)

library(data.table)

library(MCPcounter)

library(GSVA)

library(clusterProfiler)

library(estimate)

library(dplyr)

library(tidyverse)

library(pRRophetic)

library(SimDesign)

library(enrichplot)

library(forestplot)

library(e1071)

library(glmnet)

library(randomSurvivalForest)

library(randomForestSRC)

library(VennDiagram)

library(dendextend)

library(viridis)

library(pRRophetic)

library(impute)

library(SimDesign)

source(file = file.path(comRFun.path,"annTrackScale.R"))

source(file = file.path(comRFun.path,"compClinvar.R"))

source(file = file.path(comRFun.path,"batchPCA.R"))

source(file = file.path(comRFun.path,"msvmRFE.R"))

# customized functions

display.progress = function (index, totalN, breakN=20) {

if ( index %% ceiling(totalN/breakN) ==0 ) {

cat(paste(round(index*100/totalN), "% ", sep=""))

}

}

standarize.fun <- function(indata=NULL, halfwidth=NULL, centerFlag=T, scaleFlag=T) {

outdata=t(scale(t(indata), center=centerFlag, scale=scaleFlag))

if (!is.null(halfwidth)) {

outdata[outdata>halfwidth]=halfwidth

outdata[outdata<(-halfwidth)]= -halfwidth

}

return(outdata)

}

gmt2list <- function(annofile){

if (!file.exists(annofile)) {

stop("There is no such gmt file.")

}

if (tools::file_ext(annofile) == "xz") {

annofile <- xzfile(annofile)

x <- scan(annofile, what="", sep="\n", quiet=TRUE)

close(annofile)

} else if (tools::file_ext(annofile) == "gmt") {

x <- scan(annofile, what="", sep="\n", quiet=TRUE)

} else {

stop ("Only gmt and gmt.xz are accepted for gmt2list")

}

y <- strsplit(x, "\t")

names(y) <- sapply(y, `[[`, 1)

annoList <- lapply(y, `[`, c(-1,-2))

}

countToFpkm <- function(counts, effLen){

N <- sum(counts)

exp( log(counts) + log(1e9) - log(effLen) - log(N) )

}

fpkmToTpm <- function(fpkm)

{

exp(log(fpkm) - log(sum(fpkm)) + log(1e6))

}

# set colors

blue <- "#5bc0eb"

yellow <- "#fde74c"

green <- "#9bc53d"

red <- "#f25f5c"

purple <- "#531f7a"

grey <- "#8693ab"

orange <- "#fa7921"

white <- "#f2d7ee"

darkred <- "#F2042C"

lightred <- "#FF7FBF"

lightblue <- "#B2EBFF"

darkblue <- "#1d00ff"

cherry <- "#700353"

lightgrey <- "#dcddde"

nake <- "#F8C364"

gold <- "#ECE700"

cyan <- "#00B3D0"

sun <- "#E53435"

peach <- "#E43889"

violet <- "#89439B"

soil <- "#EC7D21"

lightgreen <- "#54B642"

darkblue <- "#21498D"

darkgreen <- "#009047"

brown <- "#874118"

seagreen <- "#008B8A"

jco <- c("#2874C5","#EABF00","#868686","#C6524A","#80A7DE")

jama <- c("#3B4E55","#D69044","#44A0D5","#A94747","#81AF96","#6A6599","#80796B")

npg <- c("#E64B35","#4DBBD5","#00A087","#3C5488","#F39B7F")

heatmap.BlWtRd <- c("#6699CC","white","#FF3C38")

heatmap.YlGnPe <- c("#440259","#345F8C","#228C8A","#78CE51","#FAE71F")

heatmap.GrWtRd <- c("#2b2d42","#8d99ae","#edf2f4","#ef233c","#d90429")

heatmap.L.BlYlRd <- c("#4281a4","#9cafb7","#ead2ac","#e6b89c","#fe938c")

heatmap.BlBkRd <- c("#54FEFF","#32ABAA","#125456","#000000","#510000","#A20000","#F30000")

heatmap.BlWtRd2 <- c("#183869","#4195C1","white","#CB5746","#62011D")

heatmap.fancy <- c("#10040A", "#2A0B35", "#4D155B", "#73215B", "#9C3558", "#C34D44", "#E07038", "#F2981C", "#F2CA51", "#FAF6A3")

clust.col <- c("#DD492E","#40548A","#32A087","#EC7D21")

heatmap.meth <- c("#183869","#4195C1","green","yellow","#CB5746","#62011D")

mycol <- brewer.pal(12, "Paired")

#-----------------#

# data processing #

# load external cohort expression and sample information

geo.path <- file.path(data.path,"GEO datasets")

# gse3467

gse3467.expr <- read.delim(file.path(geo.path,"gse3467.expr.matched.txt"),sep = "\t",row.names = 1,check.names = F,stringsAsFactors = F,header = T)

gse3467.sinfo <- read.delim(file.path(geo.path,"gse3467.sinfo.matched.txt"),sep = "\t",row.names = 1,check.names = F,stringsAsFactors = F,header = T)

gse3467.sinfo <- gse3467.sinfo[which(gse3467.sinfo$Type == "tumor"),]

gse3467.expr <- gse3467.expr[,rownames(gse3467.sinfo)]

# gse3678

gse3678.expr <- read.delim(file.path(geo.path,"gse3678.expr.matched.txt"),sep = "\t",row.names = 1,check.names = F,stringsAsFactors = F,header = T)

gse3678.sinfo <- read.delim(file.path(geo.path,"gse3678.sinfo.matched.txt"),sep = "\t",row.names = 1,check.names = F,stringsAsFactors = F,header = T)

gse3678.sinfo <- gse3678.sinfo[which(gse3678.sinfo$Type == "thyroid sample containing tumor cells"),,drop = F]

gse3678.expr <- gse3678.expr[,rownames(gse3678.sinfo)]

# gse27155

gse27155.expr <- read.delim(file.path(geo.path,"gse27155.expr.matched.txt"),sep = "\t",row.names = 1,check.names = F,stringsAsFactors = F,header = T)

gse27155.sinfo <- read.delim(file.path(geo.path,"gse27155.sinfo.matched.txt"),sep = "\t",row.names = 1,check.names = F,stringsAsFactors = F,header = T)

gse27155.sinfo <- gse27155.sinfo[which(gse27155.sinfo$Source != "Normal Thyroid"),,drop = F]

gse27155.expr <- gse27155.expr[,rownames(gse27155.sinfo)]

# gse33630

gse33630.expr <- read.delim(file.path(geo.path,"gse33630.expr.matched.txt"),sep = "\t",row.names = 1,check.names = F,stringsAsFactors = F,header = T)

gse33630.sinfo <- read.delim(file.path(geo.path,"gse33630.sinfo.matched.txt"),sep = "\t",row.names = 1,check.names = F,stringsAsFactors = F,header = T)

gse33630.sinfo <- gse33630.sinfo[which(gse33630.sinfo$NormalOrTumoral == "Tumor"),]

gse33630.expr <- gse33630.expr[,rownames(gse33630.sinfo)]

# gse60542

gse60542.expr <- read.delim(file.path(geo.path,"gse60542.expr.matched.txt"),sep = "\t",row.names = 1,check.names = F,stringsAsFactors = F,header = T)

gse60542.sinfo <- read.delim(file.path(geo.path,"gse60542.sinfo.matched.txt"),sep = "\t",row.names = 1,check.names = F,stringsAsFactors = F,header = T)

gse60542.sinfo <- gse60542.sinfo[which(gse60542.sinfo$Source %in% c("Papillary thyroid carcinoma")),]

gse60542.expr <- gse60542.expr[,rownames(gse60542.sinfo)]

# select GPL96 affymatrix

geo.gpl96.expr <- gse27155.expr

geo.gpl96.sinfo <- gse27155.sinfo

# combine GPL570 affymatrix

comgene.gpl570 <- intersect(rownames(gse3467.expr), rownames(gse3678.expr))

comgene.gpl570 <- intersect(comgene.gpl570, rownames(gse33630.expr))

comgene.gpl570 <- intersect(comgene.gpl570, rownames(gse60542.expr))

geo.gpl570.expr <- cbind.data.frame(gse3467.expr[comgene.gpl570,],gse3678.expr[comgene.gpl570,],gse33630.expr[comgene.gpl570,],gse60542.expr[comgene.gpl570,])

geo.gpl570.sinfo <- data.frame(row.names = c(rownames(gse3467.sinfo), rownames(gse3678.sinfo), rownames(gse33630.sinfo), rownames(gse60542.sinfo)),

Batch = rep(c("GSE3467","GSE3678","GSE33630","GSE60542"),c(nrow(gse3467.sinfo),nrow(gse3678.sinfo),nrow(gse33630.sinfo),nrow(gse60542.sinfo))))

identical(rownames(geo.gpl570.sinfo),colnames(geo.gpl570.expr))

modcombat <- model.matrix(~1, data = geo.gpl570.sinfo)

geo.gpl570.expr.combat <- as.data.frame(ComBat(dat=as.matrix(geo.gpl570.expr), batch=geo.gpl570.sinfo$Batch, mod=modcombat))

write.table(geo.gpl570.expr.combat, file = file.path(data.path,"geo.gpl570.expr.combat.txt"),sep = "\t",row.names = T,col.names = NA,quote = F)

batchPCA(indata = t(scale(t(geo.gpl570.expr))),

batch = geo.gpl570.sinfo$Batch,

fig.dir = fig.path,

PCA.fig.title = "PCA for 4 Affymetrix THCA cohorts expression profile before ComBat",

cols = npg[1:4],

showID = F,

cex = 0.9,

showLegend = T)

batchPCA(indata = t(scale(t(geo.gpl570.expr.combat))),

batch = geo.gpl570.sinfo$Batch,

fig.dir = fig.path,

PCA.fig.title = "PCA for 4 Affymetrix THCA cohorts expression profile after ComBat",

cols = npg[1:4],

showID = F,

cex = 0.9,

showLegend = T)

# load THCA phenotype information, downloaded from https://xenabrowser.net/datapages/?dataset=TCGA-THCA.GDC_phenotype.tsv&host=https%3A%2F%2Fgdc.xenahubs.net&removeHub=https%3A%2F%2Fxena.treehouse.gi.ucsc.edu%3A443

sinfo <- read.delim(file.path(data.path,"TCGA-THCA.GDC_phenotype.tsv"), sep = "\t", row.names = 1, check.names = F, stringsAsFactors = F, header = T)

# extract primary tumors of PTC

sinfo <- sinfo[substr(rownames(sinfo),14,16) == "01A",]

# load survival data from pancancer atlas, downloaded from https://gdc.cancer.gov/about-data/publications/pancanatlas

surv <- read.delim(file.path(data.path,"pancancerSurvivalData_XLu.txt"),sep = "\t",row.names = 1,check.names = F,stringsAsFactors = F,header = T)

# add survival data to sinfo

is.element(sinfo$submitter_id, rownames(surv))

sinfo$OS <- surv[sinfo$submitter_id, "OS"]

sinfo$OS.time <- surv[sinfo$submitter_id, "OS.time"]

sinfo$PFI <- surv[sinfo$submitter_id, "PFI"]

sinfo$PFI.time <- surv[sinfo$submitter_id, "PFI.time"]

sinfo <- sinfo[which(sinfo$PFI.time > 0),]

# load THCA expression data, downloaded from https://xenabrowser.net/datapages/?dataset=TCGA-THCA.htseq_fpkm.tsv&host=https%3A%2F%2Fgdc.xenahubs.net&removeHub=https%3A%2F%2Fxena.treehouse.gi.ucsc.edu%3A443

fpkm <- read.delim(file.path(data.path,"TCGA-THCA.htseq_fpkm.tsv.gz"), sep = "\t", row.names = 1, check.names = F, stringsAsFactors = F, header = T)

fpkm <- 2^fpkm - 1

# convert fpkms to tpm

tpm <- apply(fpkm, 2, fpkmToTpm)

tpm <- as.data.frame(log2(tpm + 1))

rm(fpkm); gc()

# load gene information

Ginfo <- read.delim(file.path(comAnn.path,"gencode.v22.annotation.gene.probeMap"),row.names = 1,sep = "\t",check.names = F,stringsAsFactors = F,header = T)

comgene <- intersect(rownames(Ginfo), rownames(tpm))

Ginfo <- Ginfo[comgene,]

tpm <- tpm[comgene,]

identical(rownames(tpm), rownames(Ginfo))

tpm$Gene <- Ginfo[rownames(tpm),"gene"]

tpm <- as.data.frame(apply(tpm[,setdiff(colnames(tpm), "Gene")], 2, function(x) tapply(x, INDEX = factor(tpm$Gene), FUN=median, na.rm = TRUE))) # take median value for multiple match

overlapTab <- read.table(file.path(comAnn.path,"overlapTable_hg38.txt"),sep = "\t",row.names = 1,check.names = F,stringsAsFactors = F,header = T)

Mids <- intersect(overlapTab[which(overlapTab$genetype == "protein_coding"),"genename"], rownames(tpm))

Lids <- intersect(overlapTab[which(overlapTab$genetype == "lncRNA"),"genename"], rownames(tpm))

# extract tumor and normal samples from expression data

tum.sam.expr <- colnames(tpm)[substr(colnames(tpm),14,16) == "01A"] # 497 THCA

tum.sam.expr <- intersect(rownames(sinfo), tum.sam.expr) # 496 THCA

nor.sam.expr <- colnames(tpm)[substr(colnames(tpm),14,16) == "11A"] # 56

#----------------------------#

# CAPN8 single gene analysis #

# create annotation information

annCol <- data.frame(row.names = tum.sam.expr,

Age = sinfo[tum.sam.expr,"age_at_initial_pathologic_diagnosis"],

Sex = sinfo[tum.sam.expr,"gender.demographic"],

pStage = sinfo[tum.sam.expr,"tumor_stage.diagnoses"],

tStage = sinfo[tum.sam.expr,"pathologic_T"],

nStage = sinfo[tum.sam.expr,"pathologic_N"],

mStage = sinfo[tum.sam.expr,"pathologic_M"],

Lymphnode = sinfo[tum.sam.expr,"lymph_node_preoperative_scan_indicator"],

Location = sinfo[tum.sam.expr,"primary_thyroid_gland_neoplasm_location_anatomic_site"],

OS = sinfo[tum.sam.expr,"OS"],

PFS = sinfo[tum.sam.expr,"PFI"],

stringsAsFactors = F)

annCol$Age <- ifelse(annCol$Age > median(annCol$Age), ">46","<=46")

annCol[annCol == ""] <- "Missing"

table(annCol$pStage)

annCol[which(annCol$pStage == "not reported"), "pStage"] <- "Missing"

annCol[which(annCol$pStage %in% c("stage iv","stage iva","stage ivc")), "pStage"] <- "stage iv"

table(annCol$tStage)

annCol[which(annCol$tStage %in% c("T1","T1a","T1b")), "tStage"] <- "T1"

annCol[which(annCol$tStage %in% c("T4","T4a")), "tStage"] <- "T4"

annCol[which(annCol$tStage %in% c("TX")), "tStage"] <- "Missing"

table(annCol$nStage)

annCol[which(annCol$nStage %in% c("N1","N1a","N1b")), "nStage"] <- "N1"

annCol[which(annCol$nStage %in% c("NX")), "nStage"] <- "Missing"

table(annCol$mStage)

annCol[which(annCol$mStage %in% c("MX")), "mStage"] <- "Missing"

table(annCol$Lymphnode)

table(annCol$Location)

table(annCol$OS)

table(annCol$PFS)

annCol$OS <- ifelse(annCol$OS == "1", "Dead", "Alive")

annCol$PFS <- ifelse(annCol$PFS == "1", "Progression", "Progression-free")

annCol$pStage <- factor(annCol$pStage, levels = c("stage i","stage ii","stage iii","stage iv","Missing"))

annCol$tStage <- factor(annCol$tStage, levels = c("T1","T2","T3","T4","Missing"))

annCol$nStage <- factor(annCol$nStage, levels = c("N0","N1","Missing"))

annCol$mStage <- factor(annCol$mStage, levels = c("M0","M1","Missing"))

annCol$Lymphnode <- factor(annCol$Lymphnode, levels = c("YES","NO","Missing"))

annCol$Location <- factor(annCol$Location, levels = c("Left lobe","Right lobe","Bilateral","Isthmus","Missing"))

annCol$OS <- factor(annCol$OS, levels = c("Alive","Dead"))

annCol$PFS <- factor(annCol$PFS, levels = c("Progression-free","Progression"))

annCol$CAPN8 <- as.numeric(tpm["CAPN8",rownames(annCol)])

annCol$CAPN8_Group <- ifelse(annCol$CAPN8 > median(annCol$CAPN8),"CAPN8.H","CAPN8.L")

tmp <- annCol$CAPN8; names(tmp) <- rownames(annCol)

annCol$CAPN8 <- annTrackScale(tmp, halfwidth = 2)

annColors <- list()

annColors[["Age"]] <- c("<=46" = green,">46" = orange)

annColors[["Sex"]] <- c("female" = "firebrick1", "male" = "steelblue")

annColors[["pStage"]] <- c("stage i" = mycol[2], "stage ii" = mycol[1], "stage iii" = mycol[5], "stage iv" = mycol[6], "Missing" = "white")

annColors[["tStage"]] <- c("T1" = mycol[4], "T2" = mycol[3], "T3" = mycol[7], "T4" = mycol[8], "Missing" = "white")

annColors[["nStage"]] <- c("N0" = mycol[9], "N1" = mycol[10], "Missing" = "white")

annColors[["mStage"]] <- c("M0" = mycol[11], "M1" = mycol[12], "Missing" = "white")

annColors[["Lymphnode"]] <- c("YES" = purple, "NO" = "grey90", "Missing" = "white")

annColors[["OS"]] <- c("Dead" = "black", "Alive" = "grey90")

annColors[["PFS"]] <- c("Progression" = "black", "Progression-free" = "grey90")

annColors[["Location"]] <- c("Left lobe" = npg[1], "Right lobe" = npg[2],"Bilateral" = npg[3],"Isthmus" = npg[4], "Missing" = "white")

annColors[["CAPN8"]] <- bluered(64)

annColors[["CAPN8_Group"]] <- c("CAPN8.H" = npg[1], "CAPN8.L" = npg[2])

tmp <- cbind.data.frame(sinfo[rownames(annCol),],

cluster = annCol$CAPN8_Group)

tmp$OS.time <- tmp$OS.time/30

tmp$PFI.time <- tmp$PFI.time/30

fitd <- survdiff(Surv(OS.time, OS) ~ cluster, data=tmp, na.action=na.exclude)

p.val <- 1-pchisq(fitd$chisq, length(fitd$n)-1)

fit <- survfit(Surv(OS.time, OS)~ cluster, data=tmp, type="kaplan-meier", error="greenwood", conf.type="plain", na.action=na.exclude)

names(fit$strata) <- gsub("cluster=", "", names(fit$strata))

p <- ggsurvplot(fit, conf.int=F,risk.table=T, risk.table.col="strata",palette = npg[1:2],

pval = T,data=tmp,size=1,

tables.height = 0.3,surv.median.line = "hv",

xlab = "Time (Months)",ylab = "OS",

ylim = c(0.5,1),

break.x.by = 12,

xlim = c(0,120),

risk.table.y.text = F)

p.lab <- paste0("P",

ifelse(p.val < 0.001, " < 0.001",

paste0(" = ",round(p.val, 3))))

p$plot <- p$plot + annotate("text",

x = 0, y = 0.55,

hjust = 0,

fontface = 4,

label = p.lab)

pdf.options(reset = TRUE, onefile = FALSE)

pdf(file.path(fig.path,"km of os using capn8 group in thca of tcga.pdf"),width = 5,height = 5)

print(p)

dev.off()

fitd <- survdiff(Surv(PFI.time, PFI) ~ cluster, data=tmp, na.action=na.exclude)

p.val <- 1-pchisq(fitd$chisq, length(fitd$n)-1)

fit <- survfit(Surv(PFI.time, PFI)~ cluster, data=tmp, type="kaplan-meier", error="greenwood", conf.type="plain", na.action=na.exclude)

names(fit$strata) <- gsub("cluster=", "", names(fit$strata))

p <- ggsurvplot(fit, conf.int=F,risk.table=T, risk.table.col="strata",palette = npg[1:2],

pval = T,data=tmp,size=1,

tables.height = 0.3,surv.median.line = "hv",

xlab = "Time (Months)",ylab = "PFS",

ylim = c(0.5,1),

break.x.by = 12,

xlim = c(0,120),

risk.table.y.text = F)

p.lab <- paste0("P",

ifelse(p.val < 0.001, " < 0.001",

paste0(" = ",round(p.val, 3))))

p$plot <- p$plot + annotate("text",

x = 0, y = 0.55,

hjust = 0,

fontface = 4,

label = p.lab)

pdf.options(reset = TRUE, onefile = FALSE)

pdf(file.path(fig.path,"km of pfs using capn8 group in thca of tcga.pdf"),width = 5,height = 5)

print(p)

dev.off()

# differential expression analysis

pd <- data.frame(Samples = rownames(annCol),

Group = annCol$CAPN8_Group,

stringsAsFactors = FALSE)

pd <- na.omit(pd)

design <-model.matrix(~ -1 + factor(pd$Group, levels = c("CAPN8.H","CAPN8.L")))

colnames(design) <- c("CAPN8.H","CAPN8.L")

gset <- tpm[,pd$Samples]

gset <- gset[rowSums(gset) > 0,]

fit <- limma::lmFit(gset, design = design);

contrastsMatrix <- limma::makeContrasts(CAPN8.H - CAPN8.L, levels = c("CAPN8.H", "CAPN8.L"))

fit2 <- limma::contrasts.fit(fit, contrasts = contrastsMatrix)

fit2 <- limma::eBayes(fit2, robust = T)

resData <- limma::topTable(fit2, adjust = "fdr", sort.by = "B", number = 100000, confint = T)

resData <- as.data.frame(subset(resData, select=c("logFC","t","B","P.Value","adj.P.Val","CI.L","CI.R")))

resData$id <- rownames(resData)

colnames(resData) <- c("log2fc","t","B","pvalue","padj","CI.L","CI.R","id")

resData$fc <- 2^resData$log2fc

resData <- resData[order(resData$padj),c("id","fc","log2fc","pvalue","padj","CI.L","CI.R")]

write.table(resData,file.path(res.path,"limma results of mrna between high and low group of capn8 in tcga thca.txt"),sep = "\t",row.names = F,col.names = T,quote = F)

# gsea analysis using GO BP/HALLMARK

MSIGDB.GOBP <- file.path(comAnn.path,"c5.go.bp.v7.5.1.symbols.gmt")

tmp <- read.gmt(MSIGDB.GOBP)

MSIGDB.HALLMARK <- file.path(comAnn.path,"h.all.v7.5.1.symbols.gmt")

tmp <- read.gmt(MSIGDB.HALLMARK)

geneList <- resData$log2fc; names(geneList) <- resData$id

geneList <- sort(geneList,decreasing = T)

gsea.capn8 <- GSEA(geneList = geneList,

TERM2GENE = tmp,

pvalueCutoff = 1,

seed = T,

verbose = F)

gsea.capn8.df <- as.data.frame(gsea.capn8)

#write.csv(gsea.capn8.df,file.path(res.path,"gsea gobp between high and low group of capn8 in tcga thca.csv"),row.names = F,quote = F)

write.csv(gsea.capn8.df,file.path(res.path,"gsea hallmark between high and low group of capn8 in tcga thca.csv"),row.names = F,quote = F)

gsea.capn8.df <- gsea.capn8.df[which(gsea.capn8.df$p.adjust < 0.05),]

range(gsea.capn8.df$NES)

gsea.capn8.id <- rownames(gsea.capn8.df)

# perform gsva to these significant pathways

#gobp.list <- gmt2list(MSIGDB.GOBP)

gobp.list <- gmt2list(MSIGDB.HALLMARK)

gobp.list <- gobp.list[gsea.capn8.id]

gobp.gsva.tcga <- gsva(as.matrix(tpm[,rownames(annCol)]), gobp.list, method = "gsva")

# volcano plot

x <- resData

#plot_mode <- "classic" #经典版

plot_mode <- "advanced" #酷炫版

logFCcut <- log2(1.5) #log2-foldchange

adjPcut <- 0.05 #adj.P.value

#置x，y軸的最大最小位置

xmin <- (range(x$log2fc)[1]- (range(x$log2fc)[1]+ 10))

xmax <- (range(x$log2fc)[1]+ (10-range(x$log2fc)[1]))

ymin <- 0

ymax <- 30

# 複雜的的setting for size

n1 <- length(x[, 1])

size <- rep(2, n1)

cols <- rep("grey30", n1)

names(cols)<- rownames(x)

cols[x$padj < adjPcut & x$log2fc >logFCcut]<- "#FB9A99"

cols[x$padj < adjPcut & x$log2fc > 1]<- "#ED4F4F"

cols[x$padj < adjPcut & x$log2fc < -logFCcut]<- "#B2DF8A"

cols[x$padj < adjPcut & x$log2fc < -1]<- "#329E3F"

color_transparent <- adjustcolor(cols, alpha.f = 0.8)

x$color_transparent <- color_transparent

size[x$padj < adjPcut & x$log2fc > logFCcut]<- 4

size[x$padj < adjPcut & x$log2fc > 1]<- 6

size[x$padj < adjPcut & x$log2fc < -logFCcut]<- 4

size[x$padj < adjPcut & x$log2fc < -1]<- 6

# Construct the plot object

p1 <- ggplot(data=x, aes(log2fc, -log10(padj))) +

geom_point(alpha = 0.6, size = size, colour = x$color_transparent) +

labs(x="log2FoldChange", y="-log10FDR", title="") +

ylim(c(ymin,ymax)) +

scale_x_continuous(

breaks = c(-3, -2,-1, 0, 1, 2,3), #刻度线的位置

labels = c(-3, -2,-1, 0, 1, 2,3),

limits = c(-3, 3) #x轴范围，两侧对称才好看

) +

#或用下面这行：

#xlim(c(xmin, xmax)) +

#画阈值分界线

geom_vline(xintercept = c(-1,-logFCcut, logFCcut,1), color="grey40",

linetype="longdash", lwd = 0.5) + #虚线的形状和粗细

geom_hline(yintercept = -log10(adjPcut), color="grey40",

linetype="longdash", lwd = 0.5) +

theme_bw(base_size = 12#, base_family = "Times" #修改字体

) +

theme(panel.grid=element_blank(),

axis.text = element_text(size = 12, color = "black"))

p1

ggsave(file.path(fig.path,"volcano plot of differential genes in tcga cohort.pdf"), width = 10,height = 10)

df <- as.data.frame(gsea.capn8); df <- df[,c("ID","NES","p.adjust")]

df$ID <- gsub("HALLMARK_","",df$ID)

df$group <- ifelse(df$NES > 0 & df$p.adjust < 0.05,"c", ifelse(df$NES < 0 & df$p.adjust < 0.05,"a","b"))

sortdf <- df[order(df$NES),]

sortdf$ID <- factor(sortdf$ID, levels = sortdf$ID)

head(sortdf)

ggplot(sortdf, aes(ID, NES, fill = group)) + geom_bar(stat = 'identity', width = 0.8) +

coord_flip() +

scale_fill_manual(values = c('palegreen3', 'snow3', 'dodgerblue4'), guide = FALSE) +

geom_text(data = subset(df, NES > 0),

aes(x=ID, y= -0.05, label= paste0(" ", ID), color = group),#bar璺熷潗鏍囪酱闂寸暀鍑洪棿闅?

size = 4, #瀛楃殑澶у皬

hjust = "outward" ) + #瀛楃殑瀵归綈鏂瑰紡

geom_text(data = subset(df, NES < 0),

aes(x=ID, y= 0.05, label=ID, color = group),

size = 4, hjust = "inward") +

scale_colour_manual(values = c("black","snow3","black"), guide = FALSE) +

xlab("") +ylab("Normalized Enrichment Score\n of CAPN8 High vs. Low")+

theme_bw() +

theme(panel.grid =element_blank(),

axis.text.x = element_text(size = 10, colour = "black"),

panel.border = element_rect(size = 0.6),

axis.line.y = element_blank(),

axis.ticks.y = element_blank(),

axis.text.y = element_blank()) #鍘婚櫎y杞?

ggsave(file.path(fig.path,"gsea plot for CAPN8 differential gene in tcga.pdf"), width = 10,height = 10)

# perform random forest and svm and lasso to select features

## SVM

# input <- as.data.frame(t(gobp.gsva.tcga))

# input <- cbind.data.frame(group = factor(annCol$CAPN8_Group, levels = c("CAPN8.H","CAPN8.L")), input)

# svmRFE(input, k = 5, halve.above = 100) # 5-fold crossvalidation

# nfold <- 5

# nrows <- nrow(input)

# set.seed(20000112)

# folds <- rep(1:nfold, len=nrows)[sample(nrows)]

# folds <- lapply(1:nfold, function(x) which(folds == x))

# results <- lapply(folds, svmRFE.wrap, input, k = 5, halve.above = 100) #特征选择

# top.features <- WriteFeatures(results, input, save = F)

# head(top.features)

## RF

tmp <- cbind.data.frame(sinfo[rownames(annCol),c("PFI","PFI.time")],

as.data.frame(t(gobp.gsva.tcga)))

tmp <- as.data.frame(na.omit(tmp))

set.seed(20000112)

res.rsf <- rfsrc(Surv(PFI.time, PFI) ~ ., tmp, nodesize = 20, proximity=T, tree.err = T,

forest = T, ntree = 1000, splitrule = "logrank", importance = TRUE)

set.seed(20000112)

vars <- var.select(object = res.rsf,

cause = 1,

method = "md",

conservative = c("low"),

ntree = 1000,

nodesize = 20, splitrule = "logrank",

nsplit = 10,

xvar.wt = NULL,

refit = T, fast = T,

na.action = c("na.impute"),

always.use = NULL, nrep = 10,

prefit = list(action = T, ntree = 1000,

nodesize = 20, nsplit = 10),

verbose = TRUE)

pdf(file.path(fig.path,"plot.rfsrc1.pdf"),width = 5,height = 5)

par(bty="o", mgp = c(1.9,.33,0), mar=c(4.1,4.1,2.1,2.1)+.1, las=1, tcl=-.25,xpd=F)

plot.rfsrc(res.rsf, plots.one.page = F)

invisible(dev.off())

pdf(file.path(fig.path,"plot.rfsrc2.pdf"),width = 10,height = 5)

par(bty="o", mgp = c(1.9,.33,0), mar=c(4.1,4.1,2.1,2.1)+.1, las=1, tcl=-.25,xpd=F)

plot.rfsrc(res.rsf, plots.one.page = F)

invisible(dev.off())

pdf(file.path(fig.path,"plot.variable.rfsrc.pdf"),width = 10,height = 4)

par(mgp = c(1.5,.33,0), mar=c(3,3,2,2), las=1, tcl=-.25,las = 1)

plot.variable(vars$rfsrc.refit.obj,

xvar.names = vars$topvars[1:10],

partial = F)

invisible(dev.off())

rf_fea <- vars$topvars

# res.trc <-c()

# res.trcoob<-c()

# res.testc <-c()

# topvars <- list()

# set.seed(20000112)

# for (j in 1:1000) {

# print(paste("trying for",j,"times"))

# vars <- var.select(object=res.rsf,

# cause =1,

# method = "md",

# conservative = c("medium"),

# ntree = 1000,

# nodesize = 20, splitrule = "logrankscore",

# nsplit = 10,

# xvar.wt = NULL,

# refit = T, fast = T,

# na.action = c("na.impute"),

# always.use = NULL, nrep = 10,

# prefit = list(action = T, ntree = 1000,

# nodesize = 20, nsplit = 10),

# verbose = TRUE)

#

# trc<-rcorr.cens(-vars$rfsrc.refit.obj$predicted,

# Surv(tmp$OS.time, tmp$OS))["C Index"]

# trcoob<-rcorr.cens(-vars$rfsrc.refit.obj$predicted.oob,

# Surv(tmp$OS.time, tmp$OS))["C Index"]

#

# res.trc <-rbind(res.trc, trc)

# res.trcoob <-rbind(res.trcoob, trcoob)

# if(length(vars$topvars) == 0) {

# topvars[[j]] <- "[Not Available]"

# } else {

# topvars[[j]] <- vars$topvars

# }

# }

# result<-data.frame(res.trc,res.trcoob,row.names = 1:nrow(res.trc))

# colnames(result)<-c("res.trc.cindex","res.trcoob.cindex")

# bestresult <- result[result$res.trcoob.cindex==max(result$res.trcoob.cindex),]

# bestvars <- unique(topvars[[as.numeric(rownames(bestresult))]])

# rsf.res <- cbind(tmp[,1:2],tmp[,as.character(bestvars)])

# lasso

set.seed(seed = 20000112)

ridge1_cv <- cv.glmnet(x = as.matrix(t(gobp.gsva.tcga)),

y = Surv(sinfo[rownames(annCol),"PFI.time"],sinfo[rownames(annCol),"PFI"]),

# type.measure: loss to use for cross-validation.

# type.measure = "mse",

# K = 10 is the default.

family = "cox",

nfold = 10,

# ‘alpha = 1??? is the lasso penalty, and ‘alpha = 0??? the ridge penalty.

alpha = 0)

best_ridge_coef <- as.numeric(coef(ridge1_cv, s = ridge1_cv$lambda.min))

set.seed(seed = 20000112)

cvfit <- cv.glmnet(x = as.matrix(t(gobp.gsva.tcga)),

y = Surv(sinfo[rownames(annCol),"PFI.time"],sinfo[rownames(annCol),"PFI"]),

family = "cox",

# type.measure = "deviance",

alpha = 1,

penalty.factor = 1 / abs(best_ridge_coef),

# keep = T,

nfold = 10)

myCoefs <- coef(cvfit, s="lambda.min")

#lasso_fea <- myCoefs@Dimnames[[1]][which(myCoefs != 0 )]

lasso_fea <- rownames(coef(cvfit, s = 'lambda.min'))[coef(cvfit, s = 'lambda.min')[,1]!= 0] ### returns nonzero coefs

pdf(file.path(fig.path,"lasso.pdf"),width = 4.5,height = 4)

par(bty="o", mgp = c(1.9,.33,0), mar=c(4.1,4.1,2.1,2.1)+.1, las=1, tcl=-.25,xpd=F)

plot(cvfit$glmnet.fit, "lambda", label=F)

abline(v=log(cvfit$lambda.min),lwd=2,col="black",lty=4) # 1.09

invisible(dev.off())

pdf(file.path(fig.path,"cvfit.pdf"),width = 4.5,height = 4)

par(bty="o", mgp = c(1.9,.33,0), mar=c(4.1,4.1,2.1,2.1)+.1, las=1, tcl=-.25)

plot(cvfit)

abline(h=min(cvfit$cvm),lwd=2,col="black",lty=4)

points(log(cvfit$lambda.min),min(cvfit$cvm),pch=18,cex=2,col="black")

points(log(cvfit$lambda.min),min(cvfit$cvm),pch=18,cex=1.5,col="skyblue")

invisible(dev.off())

# select common feature identified from lasso and rf

com_fea <- intersect(lasso_fea, rf_fea)

venn.diagram(

x = list(lasso_fea,rf_fea),

category.names = c("Adaptive LASSO","Random forest"),

filename = file.path(fig.path, "selected HALLMARK pathways.tiff"),

main = "",

output = TRUE,

# Output features

imagetype = "tiff" ,

height = 1500 ,

width = 1500 ,

resolution = 500,

compression = "lzw",

# Circles

lwd = 2,

lty = 'blank',

fill = c("#C8EAB0","#3A8FC0"),

# Numbers

cex = 1,

fontface = "bold",

#fontfamily = "arial",

# Set names

cat.cex = 0.2,

cat.fontface = "bold",

cat.default.pos = "outer")

# use PFS to further filtering pathway

tmp <- cbind.data.frame(sinfo[rownames(annCol),c("PFI","PFI.time")],

as.data.frame(t(gobp.gsva.tcga[c(com_fea),])))

tmp <- as.data.frame(na.omit(tmp))

tmp$PFI.time <- tmp$PFI.time/30

for(i in 3:ncol(tmp)){

display.progress(index = i,totalN = ncol(tmp),breakN = 20)

bp <- colnames(tmp)[i]

group <- ifelse(tmp[,i] > median(tmp[,i]),"High","Low")

group <- factor(group, levels = c("High","Low"))

fitd <- survdiff(Surv(PFI.time, PFI) ~ group, data=tmp, na.action=na.exclude)

p.val <- 1-pchisq(fitd$chisq, length(fitd$n)-1)

fit <- survfit(Surv(PFI.time, PFI)~ group, data=tmp, type="kaplan-meier", error="greenwood", conf.type="plain", na.action=na.exclude)

names(fit$strata) <- gsub("group=", "", names(fit$strata))

p <- ggsurvplot(fit, conf.int=F,risk.table=T, risk.table.col="strata",palette = npg[1:2],

pval = T,data=tmp,size=1,

title = bp,

tables.height = 0.3,surv.median.line = "hv",

xlab = "Time (Months)",ylab = "PFS",

ylim = c(0.5,1),

break.x.by = 12,

xlim = c(0,120),

risk.table.y.text = F)

p.lab <- paste0("P",

ifelse(p.val < 0.001, " < 0.001",

paste0(" = ",round(p.val, 3))))

p$plot <- p$plot + annotate("text",

x = 0, y = 0.55,

hjust = 0,

fontface = 4,

label = p.lab)

pdf.options(reset = TRUE, onefile = FALSE)

pdf(file.path(fig.path,paste0("km of pfs using ",bp," group in thca of tcga.pdf")),width = 5,height = 5)

print(p)

dev.off()

}

#---------------------------------------------#

# use E2F targets genes to perform clustering #

#e2f.targets <- gobp.list$HALLMARK_E2F_TARGETS

e2f.targets <- gsea.capn8.df["HALLMARK_E2F_TARGETS","core_enrichment"]

e2f.targets <- unlist(strsplit(e2f.targets,"/", fixed = T))

indata <- t(scale(t(tpm[intersect(rownames(tpm),e2f.targets),rownames(annCol)])))

hcs <- hclust(distanceMatrix(as.matrix(indata), "euclidean"), "ward.D")

hcs <- click_rotate(as.dendrogram(hcs))

hcs <- as.hclust(hcs)

hcs.thca <- hcs

hcg <- hclust(distanceMatrix(as.matrix(t(indata)), "pearson"), "ward.D")

group <- cutree(hcs.thca, k = 2)

group <- paste0("CS",group); names(group) <- colnames(indata)

annCol$E2F.Clust <- group

annColors[["E2F.Clust"]] <- c("CS1" = jco[2], "CS2" = jco[1])

plotdata <- standarize.fun(indata, halfwidth = 2)

hm <- pheatmap(plotdata,

cluster_rows = hcg,

cluster_cols = hcs.thca,

use_raster = F,

show_colnames = F,

show_rownames = F,

color = NMF:::ccRamp(heatmap.BlBkRd,64),

border_color = NA,

name = "Expr.",

cellwidth = 400/ncol(plotdata),

cellheight = 300/nrow(plotdata),

annotation_col = annCol[colnames(plotdata),c("E2F.Clust","PFS","Age","Sex","tStage","nStage","mStage","pStage","Lymphnode","Location","CAPN8","CAPN8_Group")],

annotation_colors = annColors[c("E2F.Clust","PFS","Age","Sex","tStage","nStage","mStage","pStage","Lymphnode","Location","CAPN8","CAPN8_Group")])

pdf(file.path(fig.path,"unsupervised heatmap of e2f targets genes in tcga thca.pdf"), width = 8,height = 10)

draw(hm, heatmap_legend_side = "bottom",annotation_legend_side = "bottom")

invisible(dev.off())

tmp <- cbind.data.frame(sinfo[rownames(annCol),c("PFI","PFI.time")],

group = annCol$E2F.Clust)

tmp <- as.data.frame(na.omit(tmp))

tmp$PFI.time <- tmp$PFI.time/30

fitd <- survdiff(Surv(PFI.time, PFI) ~ group, data=tmp, na.action=na.exclude)

p.val <- 1-pchisq(fitd$chisq, length(fitd$n)-1)

fit <- survfit(Surv(PFI.time, PFI)~ group, data=tmp, type="kaplan-meier", error="greenwood", conf.type="plain", na.action=na.exclude)

names(fit$strata) <- gsub("group=", "", names(fit$strata))

p <- ggsurvplot(fit, conf.int=F,risk.table=T, risk.table.col="strata",palette = jco[2:1],

pval = T,data=tmp,size=1,

tables.height = 0.3,surv.median.line = "hv",

xlab = "Time (Months)",ylab = "PFS",

ylim = c(0.5,1),

break.x.by = 12,

xlim = c(0,120),

risk.table.y.text = F)

p.lab <- paste0("P",

ifelse(p.val < 0.001, " < 0.001",

paste0(" = ",round(p.val, 3))))

p$plot <- p$plot + annotate("text",

x = 0, y = 0.55,

hjust = 0,

fontface = 4,

label = p.lab)

pdf.options(reset = TRUE, onefile = FALSE)

pdf(file.path(fig.path,"km of pfs using E2F cluster in thca of tcga.pdf"),width = 5,height = 5)

print(p)

dev.off()

#-------------------------#

# subtype characterzation #

mcp.ptc <- MCPcounter.estimate(as.matrix(tpm[,tum.sam.expr]),

featuresType = "HUGO_symbols",

genes = read.table(file.path(comAnn.path,"genes.txt"),sep = "\t",stringsAsFactors = F,header = T,colClasses = "character",check.names = F),

probesets = read.table(file.path(comAnn.path,"probesets.txt"),sep = "\t",stringsAsFactors = F,header = F,colClasses = "character",check.names = F))

indata <- tpm[,tum.sam.expr]

write.table(indata,file = file.path(res.path,"TCGA_log2TPM_hugo.txt"),sep = "\t",row.names = T,col.names = NA,quote = F)

filterCommonGenes(input.f=file.path(res.path, "TCGA_log2TPM_hugo.txt") , output.f=file.path(res.path,"TCGA_log2TPM_hugo_ESTIMATE.txt"), id="GeneSymbol")

estimateScore(file.path(res.path,"TCGA_log2TPM_hugo_ESTIMATE.txt"), file.path(res.path,"TCGA_log2TPM_hugo_estimate_score.txt"), platform="affymetrix")

est.tcga <- read.table(file = file.path(res.path,"TCGA_log2TPM_hugo_estimate_score.txt"),header = T,row.names = NULL,check.names = F,stringsAsFactors = F,sep = "\t")

rownames(est.tcga) <- est.tcga[,2]; colnames(est.tcga) <- est.tcga[1,]; est.tcga <- est.tcga[-1,c(-1,-2)];

est.tcga <- sapply(est.tcga, as.numeric); rownames(est.tcga) <- c("StromalScore","ImmuneScore","ESTIMATEScore","TumorPurity"); est.tcga.backup = as.data.frame(est.tcga); colnames(est.tcga.backup) <- colnames(indata)

est.tcga <- annTrackScale(indata = est.tcga, halfwidth = 2, poolsd = F); est.tcga <- as.data.frame(t(est.tcga))

rownames(est.tcga) <- tum.sam.expr

annCol$IES <- est.tcga[rownames(annCol),"ImmuneScore"]

annCol$SES <- est.tcga[rownames(annCol),"StromalScore"]

annColors[["IES"]] <- annColors[["SES"]] <- bluered(64)

imm.targets <- c("CD274","PDCD1","CD247","PDCD1LG2","CTLA4","TNFRSF9","TNFRSF4","TLR9")

group2 <- cutree(hcs.thca, k = 4)

group2 <- paste0("CS",group2); names(group2) <- rownames(annCol)

annCol$ImmClust <- group2

annCol[which(annCol$ImmClust == "CS4"),"ImmClust"] <- "CS2"

annCol[which(annCol$ImmClust == "CS3"),"ImmClust"] <- "CS4"

annCol[which(annCol$ImmClust == "CS2"),"ImmClust"] <- "CS3"

annCol[which(annCol$ImmClust == "CS4"),"ImmClust"] <- "CS2"

annColors[["ImmClust"]] <- c("CS1" = npg[1], "CS2" = npg[2], "CS3" = npg[3])

plotdata <- standarize.fun(tpm[imm.targets,rownames(annCol)],halfwidth = 2)

hm1 <- pheatmap(plotdata,

border_color = NA,

show_rownames = TRUE,

show_colnames = F,

#use_raster = FALSE,

cluster_rows = FALSE,

cluster_cols = hcs.thca,

cellheight = 12,

cutree_cols = 2,

cellwidth = 250/ncol(plotdata),

name = "Immunotherapy targets",

annotation_col = annCol[colnames(plotdata),c("ImmClust","E2F.Clust","IES","SES","OS","PFS")],

annotation_colors = annColors[c("ImmClust","E2F.Clust","IES","SES","OS","PFS")],

color = colorpanel(64,low=blue,mid = "black",high=gold))

plotdata <- standarize.fun(mcp.ptc[,rownames(annCol)],halfwidth = 2)

hm2 <- pheatmap(plotdata,

border_color = NA,

show_rownames = TRUE,

show_colnames = F,

#use_raster = FALSE,

cluster_rows = FALSE,

cluster_cols = hcs.thca,

cellheight = 12,

cutree_cols = 2,

cellwidth = 250/ncol(plotdata),

name = "MCPcounter",

color = greenred(64))

pdf(file.path(fig.path,"heatmap of refinement of E2F cluster to immclust in tcga.pdf"), width = 6,height = 10)

draw(hm1 %v% hm2, heatmap_legend_side = "bottom", annotation_legend_side = "bottom")

invisible(dev.off())

tmp <- cbind.data.frame(sinfo[rownames(annCol),c("PFI","PFI.time")],

group = annCol$ImmClust)

tmp <- as.data.frame(na.omit(tmp))

tmp$PFI.time <- tmp$PFI.time/30

fitd <- survdiff(Surv(PFI.time, PFI) ~ group, data=tmp, na.action=na.exclude)

p.val <- 1-pchisq(fitd$chisq, length(fitd$n)-1)

fit <- survfit(Surv(PFI.time, PFI)~ group, data=tmp, type="kaplan-meier", error="greenwood", conf.type="plain", na.action=na.exclude)

names(fit$strata) <- gsub("group=", "", names(fit$strata))

p <- ggsurvplot(fit, conf.int=F,risk.table=T, risk.table.col="strata",palette = npg[1:3],

pval = T,data=tmp,size=1,

tables.height = 0.3,surv.median.line = "hv",

xlab = "Time (Months)",ylab = "PFS",

ylim = c(0.5,1),

break.x.by = 12,

xlim = c(0,120),

risk.table.y.text = F)

p.lab <- paste0("P",

ifelse(p.val < 0.001, " < 0.001",

paste0(" = ",round(p.val, 3))))

p$plot <- p$plot + annotate("text",

x = 0, y = 0.55,

hjust = 0,

fontface = 4,

label = p.lab)

pdf.options(reset = TRUE, onefile = FALSE)

pdf(file.path(fig.path,"km of pfs using immclust in thca of tcga.pdf"),width = 5,height = 5)

print(p)

dev.off()

my_comparisons <- list( c("CS1", "CS2"),

c("CS2", "CS3"),

c("CS1", "CS3"))

ggplot(data = annCol,aes(x = ImmClust,

y = IES,

fill = ImmClust))+

scale_fill_manual(values = npg[1:3]) +

geom_violin(alpha = 0.4, position = position_dodge(width = .75),

size = 0.8, color="black") +

geom_boxplot(notch = FALSE, outlier.size = -1,

color="black", lwd=0.8, alpha = 0.7) +

geom_point(shape = 21, size=2,

position = position_jitterdodge(),

color="black", alpha = 1) +

theme_bw() +

ylab("IES") +

xlab("") +

theme(axis.text.x = element_text(hjust = 1, size = 12, color = "black"),

axis.ticks = element_line(size=0.2, color="black"),

axis.ticks.length = unit(0.2, "cm"),

legend.position = "top",

panel.background = element_blank(),

panel.grid = element_blank(),

axis.title = element_text(size = 12),

axis.text = element_text(size = 12)) +

stat_compare_means(comparisons = my_comparisons,method = "wilcox.test") +

stat_compare_means(method = "kruskal.test", label.y = -2.5)

ggsave(file.path(fig.path,"boxplot for immune enrichment score in immclust of tcga.pdf"),width = 4,height = 5)

ggplot(data = annCol,aes(x = ImmClust,

y = SES,

fill = ImmClust))+

scale_fill_manual(values = npg[1:3]) +

geom_violin(alpha = 0.4, position = position_dodge(width = .75),

size = 0.8, color="black") +

geom_boxplot(notch = FALSE, outlier.size = -1,

color="black", lwd=0.8, alpha = 0.7) +

geom_point(shape = 21, size=2,

position = position_jitterdodge(),

color="black", alpha = 1) +

theme_bw() +

ylab("SES") +

xlab("") +

theme(axis.text.x = element_text(hjust = 1, size = 12, color = "black"),

axis.ticks = element_line(size=0.2, color="black"),

axis.ticks.length = unit(0.2, "cm"),

legend.position = "top",

panel.background = element_blank(),

panel.grid = element_blank(),

axis.title = element_text(size = 12),

axis.text = element_text(size = 12)) +

stat_compare_means(comparisons = my_comparisons,method = "wilcox.test") +

stat_compare_means(method = "kruskal.test", label.y = -2.5)

ggsave(file.path(fig.path,"boxplot for stronal enrichment score in immclust of tcga.pdf"),width = 4,height = 5)

ggplot(data = annCol,aes(x = ImmClust,

y = scale(as.numeric(tpm["CAPN8",rownames(annCol)])),

fill = ImmClust))+

scale_fill_manual(values = npg[1:3]) +

geom_violin(alpha = 0.4, position = position_dodge(width = .75),

size = 0.8, color="black") +

geom_boxplot(notch = FALSE, outlier.size = -1,

color="black", lwd=0.8, alpha = 0.7) +

geom_point(shape = 21, size=2,

position = position_jitterdodge(),

color="black", alpha = 1) +

theme_bw() +

ylab("log2(TPM + 1) of CAPN8\n[z-scored]") +

xlab("") +

theme(axis.text.x = element_text(hjust = 1, size = 12, color = "black"),

axis.ticks = element_line(size=0.2, color="black"),

axis.ticks.length = unit(0.2, "cm"),

legend.position = "top",

panel.background = element_blank(),

panel.grid = element_blank(),

axis.title = element_text(size = 12),

axis.text = element_text(size = 12)) +

stat_compare_means(comparisons = my_comparisons,method = "wilcox.test") +

stat_compare_means(method = "kruskal.test", label.y = -2.5)

ggsave(file.path(fig.path,"boxplot for capn8 expression in immclust of tcga.pdf"),width = 4,height = 5)

ggplot(data = annCol,aes(x = E2F.Clust,

y = scale(as.numeric(tpm["CAPN8",rownames(annCol)])),

fill = E2F.Clust))+

scale_fill_manual(values = jco[2:1]) +

geom_violin(alpha = 0.4, position = position_dodge(width = .75),

size = 0.8, color="black") +

geom_boxplot(notch = FALSE, outlier.size = -1,

color="black", lwd=0.8, alpha = 0.7) +

geom_point(shape = 21, size=2,

position = position_jitterdodge(),

color="black", alpha = 1) +

theme_bw() +

ylab("log2(TPM + 1) of CAPN8\n[z-scored]") +

xlab("") +

theme(axis.text.x = element_text(hjust = 1, size = 12, color = "black"),

axis.ticks = element_line(size=0.2, color="black"),

axis.ticks.length = unit(0.2, "cm"),

legend.position = "top",

panel.background = element_blank(),

panel.grid = element_blank(),

axis.title = element_text(size = 12),

axis.text = element_text(size = 12)) +

stat_compare_means(method = "kruskal.test", label.y = -2.5)

ggsave(file.path(fig.path,"boxplot for capn8 expression in E2F cluster of tcga.pdf"),width = 4,height = 5)

tcga.tide <- sweep(tpm[,rownames(annCol)],1,apply(tpm[,nor.sam.expr], 1, median))

write.table(tcga.tide,file.path(res.path,"tcga.tide.txt"),sep = "\t",row.names = T,col.names = NA,quote = F)

tcga.tide.res <- read.csv(file.path(res.path,"tcga.tide.output.csv"),row.names = 1,check.names = F,stringsAsFactors = F,header = T)

annCol$TIDE <- tcga.tide.res[rownames(annCol),"Responder"]

annCol$TIDE.score <- tcga.tide.res[rownames(annCol),"TIDE"] # higher value means higher immune evasion

table(annCol$TIDE, annCol$E2F.Clust)

table(annCol$TIDE, annCol$ImmClust)

annColors[["TIDE"]] <- c("True" = purple,"False" = "grey90")

annColors[["TIDE.score"]] <- bluered(64)

# submap to analyze immunotherapy

generateInputFileForSubMap <- function(in_gct, gct_file, cls_file, sam_info, type_name = "type"){

in_gct <- data.frame(GeneID=rownames(in_gct),

description="na",

in_gct,

stringsAsFactors = F,

check.names = F)

cat("#1.2\n", file = gct_file)

cat(nrow(in_gct),"\t",ncol(in_gct)-2,"\n", file = gct_file, append = T)

cat(paste(colnames(in_gct), collapse = "\t"),"\n", file = gct_file, append = T)

for(i in 1:nrow(in_gct)) cat(paste(in_gct[i,], collapse = "\t"),"\n", file = gct_file, append = T)

cat(nrow(sam_info),length(levels(factor(sam_info$rank))),1, "\n", file = cls_file )

cat("#", paste0(levels(factor(sam_info[, type_name])), collapse = " " ), "\n", file = cls_file, sep = "", append = T)

cat(as.numeric(factor(sam_info[, type_name])), file = cls_file, append = T)

}

# create submap format for skin melanoma

skcm.immunotherapy.logNC <- read.table(file.path(data.path,"skcm.immunotherapy.47samples.log2CountsNorm.txt"),sep = "\t",row.names = 1,header = T,check.names = F,stringsAsFactors = F) #原文提供的log2转化的标准化count值

rownames(skcm.immunotherapy.logNC) <- toupper(rownames(skcm.immunotherapy.logNC))

skcm.immunotherapy.info <- read.table(file.path(data.path,"skcm.immunotherapy.47sampleInfo.txt"),sep = "\t",row.names = 1,header = T,check.names = F,stringsAsFactors = F)

skcm.immunotherapy.info <- skcm.immunotherapy.info[order(skcm.immunotherapy.info$label),]

skcm.immunotherapy.info$rank <- rep(c(1,2,3,4),times=as.character(table(skcm.immunotherapy.info$label))) #1: CTLA4_noR 2: CTLA4_R 3:PD1_noR 4:PD1_R

# create submap format for tcga

GENELIST <- intersect(rownames(tpm),rownames(skcm.immunotherapy.logNC))

sam_info <- skcm.immunotherapy.info

in_gct <- skcm.immunotherapy.logNC[GENELIST,rownames(skcm.immunotherapy.info)]

# generate file

gct_file <- file.path(res.path,"skcm.immunotherapy.for.SubMap.gct")

cls_file <- file.path(res.path,"skcm.immunotherapy.for.SubMap.cls")

generateInputFileForSubMap(in_gct = in_gct, gct_file = gct_file, cls_file = cls_file, sam_info = sam_info, type_name = "rank")

# extract subtypes

sample.C1 <- rownames(annCol[annCol$ImmClust == "CS1",])

sample.C2 <- rownames(annCol[annCol$ImmClust == "CS2",])

sample.C3 <- rownames(annCol[annCol$ImmClust == "CS3",])

sam_info <- data.frame("ImmClust"=c(sample.C1,sample.C2,sample.C3),row.names = c(sample.C1,sample.C2,sample.C3))

sam_info$rank <- rep(c(1,2,3),times=c(length(sample.C1),length(sample.C2),length(sample.C3)))

# generate file

gct_file <- file.path(res.path,"PTC.ImmClust.for.SubMap.gct")

cls_file <- file.path(res.path,"PTC.ImmClust.for.SubMap.cls")

in_gct <- tpm[GENELIST,rownames(sam_info)]

generateInputFileForSubMap(in_gct = in_gct, gct_file = gct_file, cls_file = cls_file, sam_info = sam_info, type_name = "rank")

#----------------------------------#

# differential expression analysis #

ptc.movics <- list("clust.res" = annCol,

"mo.method" = "thca.immclust")

ptc.movics$clust.res$samID <- rownames(ptc.movics$clust.res)

ptc.movics$clust.res$clust <- sapply(ptc.movics$clust.res$ImmClust,

switch,

"CS1" = 1,

"CS2" = 2,

"CS3" = 3)

Mids.common <- intersect(Mids, rownames(geo.gpl96.expr))

Mids.common <- intersect(Mids.common, rownames(geo.gpl570.expr.combat))

runDEA(dea.method = "limma",

expr = tpm[Mids.common,rownames(annCol)], # raw count data

moic.res = ptc.movics,

res.path = res.path,

overwt = TRUE,

prefix = "TCGA-THCA") # prefix of figure name

# choose limma result to identify subtype-specific up-regulated biomarkers

marker.up.tcga <- runMarker(moic.res = ptc.movics,

clust.col = npg[1:3],

dea.method = "limma", # name of DEA method

prefix = "TCGA-THCA", # MUST be the same of argument in runDEA()

dat.path = res.path, # path of DEA files

res.path = res.path, # path to save marker files

p.cutoff = 0.05, # p cutoff to identify significant DEGs

p.adj.cutoff = 0.05, # padj cutoff to identify significant DEGs

dirct = "up", # direction of dysregulation in expression

n.marker = 10, # number of biomarkers for each subtype

doplot = TRUE, # generate diagonal heatmap

norm.expr = tpm[Mids.common,rownames(annCol)], # use normalized expression as heatmap input

annCol = annCol[,c("IES","SES","TIDE.score","TIDE")], # sample annotation in heatmap

annColors = annColors[c("IES","SES","TIDE.score","TIDE")], # colors for sample annotation

show_rownames = FALSE, # show no rownames (biomarker name)

fig.path = fig.path,

color = greenred(64),

halfwidth = 2,

width = 12,

height = 10,

fig.name = "upregulated biomarker heatmap in tcga")

#write.table(marker.up.tcga$templates,file.path(res.path,"mrna marker list for tcga thca.txt"),sep = "\t",row.names = F,col.names = T,quote = F)

# copy number variation

# copy number variation analysis

cnv <- read.table(file.path(data.path,"THCA.snp__genome_wide_snp_6__broad_mit_edu__Level_3__segmented_scna_minus_germline_cnv_hg19__seg.seg.txt"),sep = "\t",row.names = NULL,header = T,check.names = F,stringsAsFactors = F)

cnv$Sample <-substr(cnv$Sample,start = 1,stop = 16)

comsam <- intersect(cnv$Sample,rownames(annCol)) # 488

cnv <- cnv[which(cnv$Sample %in% comsam),]

write.table(as.data.frame(cnv),file.path(res.path,"ptc_488_segment_forGISTIC2.0.txt"),sep = "\t",row.names = F,quote = F)

# create marker file for GISTIC2.0

marker <- cnv[,1:4]

a <- b <- c <- c()

for(i in 1:nrow(marker)) {

a <- c(a,rep(marker[i,"Sample"],2))

b <- c(b,rep(marker[i,"Chromosome"],2))

c <- c(c,marker[i,"Start"],marker[i,"End"])

}

marker <- data.frame(Marker.Name = a,Chromosome = b,Marker.Position=c,stringsAsFactors = F)

write.table(as.data.frame(marker),file.path(res.path,"ptc_488_marker_forGISTIC2.0.txt"),sep = "\t",row.names = F,quote = F)

rm(a); rm(b); rm(c)

seg.C1.cnv <- cnv[which(cnv$Sample %in% rownames(annCol[which(annCol$ImmClust == "CS1"),])),]

seg.C2.cnv <- cnv[which(cnv$Sample %in% rownames(annCol[which(annCol$ImmClust == "CS2"),])),]

seg.C3.cnv <- cnv[which(cnv$Sample %in% rownames(annCol[which(annCol$ImmClust == "CS3"),])),]

write.table(as.data.frame(seg.C1.cnv),file.path(res.path,"ptc_immclust_cs1_segment_forGISTIC2.0.txt"),sep = "\t",row.names = F,quote = F)

write.table(as.data.frame(seg.C2.cnv),file.path(res.path,"ptc_immclust_cs2_segment_forGISTIC2.0.txt"),sep = "\t",row.names = F,quote = F)

write.table(as.data.frame(seg.C3.cnv),file.path(res.path,"ptc_immclust_cs3_segment_forGISTIC2.0.txt"),sep = "\t",row.names = F,quote = F)

# create marker file for GISTIC2.0

marker <- seg.C1.cnv[,1:4]

a <- b <- c <- c()

for(i in 1:nrow(marker)) {

a <- c(a,rep(marker[i,"Sample"],2))

b <- c(b,rep(marker[i,"Chromosome"],2))

c <- c(c,marker[i,"Start"],marker[i,"End"])

}

marker <- data.frame(Marker.Name = a,Chromosome = b,Marker.Position=c,stringsAsFactors = F)

write.table(as.data.frame(marker),file.path(res.path,"ptc_immclust_cs1_marker_forGISTIC2.0.txt"),sep = "\t",row.names = F,quote = F)

rm(a); rm(b); rm(c)

marker <- seg.C2.cnv[,1:4]

a <- b <- c <- c()

for(i in 1:nrow(marker)) {

a <- c(a,rep(marker[i,"Sample"],2))

b <- c(b,rep(marker[i,"Chromosome"],2))

c <- c(c,marker[i,"Start"],marker[i,"End"])

}

marker <- data.frame(Marker.Name = a,Chromosome = b,Marker.Position=c,stringsAsFactors = F)

write.table(as.data.frame(marker),file.path(res.path,"ptc_immclust_cs2_marker_forGISTIC2.0.txt"),sep = "\t",row.names = F,quote = F)

rm(a); rm(b); rm(c)

marker <- seg.C3.cnv[,1:4]

a <- b <- c <- c()

for(i in 1:nrow(marker)) {

a <- c(a,rep(marker[i,"Sample"],2))

b <- c(b,rep(marker[i,"Chromosome"],2))

c <- c(c,marker[i,"Start"],marker[i,"End"])

}

marker <- data.frame(Marker.Name = a,Chromosome = b,Marker.Position=c,stringsAsFactors = F)

write.table(as.data.frame(marker),file.path(res.path,"ptc_immclust_cs3_marker_forGISTIC2.0.txt"),sep = "\t",row.names = F,quote = F)

rm(a); rm(b); rm(c)

# Create a chromosomes reference objects function

chrom_extract <- function(BSgenome.hg = NULL) {

if (is.null(BSgenome.hg )) stop("NULL object !", call. = FALSE)

obj <- list(species = GenomeInfoDb::organism(BSgenome.hg), genomebuild = BSgenome::providerVersion(BSgenome.hg))

df <- data.frame(chrom = BSgenome::seqnames(BSgenome.hg), chrN = seq_along(BSgenome::seqnames(BSgenome.hg)), chr.length = GenomeInfoDb::seqlengths(BSgenome.hg), stringsAsFactors = FALSE)

df <- df[1:24,]

df$chr.length.sum <- cumsum(as.numeric(df$chr.length))

df$chr.length.cumsum <- c(0, df$chr.length.sum[-nrow(df)])

df$middle.chr <- round(diff(c(0, df$chr.length.sum)) /2)

df$middle.chr.genome <- df$middle.chr + df$chr.length.cumsum

obj$chromosomes <- df

obj$chrom2chr <- sapply(obj$chromosomes$chrom, function(k) { obj$chromosomes$chrN[obj$chromosomes$chrom == k]}, simplify = FALSE)

obj$chr2chrom <- sapply(obj$chromosomes$chrN, function(k) { obj$chromosomes$chrom[obj$chromosomes$chrN == k]}, simplify = FALSE)

names(obj$chr2chrom) <- obj$chromosomes$chrN

obj$genome.length <- sum(as.numeric(obj$chromosomes$chr.length), na.rm = TRUE)

return(obj)

}

# Extract a chromosomes reference loci

BSgenome.hg = "BSgenome.Hsapiens.UCSC.hg19"

BSg.obj <- getExportedValue(BSgenome.hg, BSgenome.hg)

genome.version <- BSgenome::providerVersion(BSg.obj)

chrom <- chrom_extract(BSg.obj)

#str(chrom)

pdf(file = file.path(fig.path,"cnv.scores.gistic.pdf"),12,12)

par(mfrow=c(4,1), mgp = c(1.5,.33,0), mar=c(4,4,3,0.1), las=1, tcl=-.25,las = 1)

### all PTC ###

scores <- read.table(file.path(res.path,"GISTIC/440600/ptc_all.scores.gistic"), sep="\t",header=T,stringsAsFactors = F)

# Important step for accurate length to match back to continual chrom loci

scores[scores$Chromosome==23,"Chromosome"]="X"

scores[scores$Chromosome==24,"Chromosome"]="Y"

chrID <- unname(unlist(chrom$chrom2chr[as.character(paste0("chr",scores$Chromosome))]))

scores$Start.geno <- scores$Start + chrom$chromosomes$chr.length.cumsum[chrID]

scores$End.geno <- scores$End + chrom$chromosomes$chr.length.cumsum[chrID]

# Prepare input data for ploting

scores.amp <- scores[scores$Type=="Amp",]

scores.amp$G.score <- scores.amp$G.score * 1

scores.del <- scores[scores$Type=="Del",]

scores.del$G.score <- scores.del$G.score * -1

scores <- rbind.data.frame(scores.amp,scores.del)

# seg.col = list(gain = "red", outscale.gain = "darkred", loss = "blue", outscale.red = "midnightblue")

ylim <- c(min(scores$G.score)-0.1,max(scores$G.score)+0.1)

title="CN gistic score of all thyroid carcinoma (n = 488)"

plot(scores.amp$Start.geno, scores.amp$G.score,

pch = ".", type='h',cex = 2, xaxs = "i", yaxs = "i", xlim = c(0,chrom$genome.length), ylim = ylim,

main = title, cex.main = 2, ylab = "gistic score", xlab = NA,

cex.lab = 2, col = adjustcolor("darkred", alpha.f = .8), xaxt = "n", lwd = 2, las=1) # las=1 rotating axis labels in R

lines(scores.del$Start.geno, scores.del$G.score, type='h', lwd = 2, col = adjustcolor("midnightblue", alpha.f = .8))

ink <- chrom$chromosomes$chrN %in% chrID

yrange = abs(diff(ylim))

m.pos <- c(ylim[1]+0.05,ylim[2]-0.05)

m.mod <- -(chrom$chromosomes$chrN[ink] %% 2) +2

try(text(x = chrom$chromosomes$middle.chr.geno[ink], y = m.pos[m.mod], labels = chrom$chromosomes$chrom[ink], cex = 1))

abline(h = 0.0, col = 1, lwd = 1, lty = 3)

abline(v = c(0,chrom$chromosomes$chr.length.sum), col = 1, lty = 3, lwd = 1)

col1 <- adjustcolor("darkred", alpha.f = .8)

col2 <- adjustcolor("midnightblue", alpha.f = .8)

# The position of the legend can be specified also using the following keywords : "bottomright", "bottom", "bottomleft", "left", "topleft", "top", "topright", "right" and "center".

legend("topleft", c("gain","loss"), cex=0.6, bty="n", fill=c(col1,col2))

### PTC CS1 ###

scores <- read.table(file.path(res.path,"GISTIC/440601/ptc_immclust_cs1.scores.gistic"), sep="\t",header=T,stringsAsFactors = F)

# Important step for accurate length to match back to continual chrom loci

scores[scores$Chromosome==23,"Chromosome"]="X"

scores[scores$Chromosome==24,"Chromosome"]="Y"

chrID <- unname(unlist(chrom$chrom2chr[as.character(paste0("chr",scores$Chromosome))]))

scores$Start.geno <- scores$Start + chrom$chromosomes$chr.length.cumsum[chrID]

scores$End.geno <- scores$End + chrom$chromosomes$chr.length.cumsum[chrID]

# Prepare input data for ploting

scores.amp <- scores[scores$Type=="Amp",]

scores.amp$G.score <- scores.amp$G.score * 1

scores.del <- scores[scores$Type=="Del",]

scores.del$G.score <- scores.del$G.score * -1

scores <- rbind.data.frame(scores.amp,scores.del)

# seg.col = list(gain = "red", outscale.gain = "darkred", loss = "blue", outscale.red = "midnightblue")

ylim <- c(min(scores$G.score)-0.1,max(scores$G.score)+0.1)

title="CN gistic score of ImmClust CS1 (n = 121)"

plot(scores.amp$Start.geno, scores.amp$G.score,

pch = ".", type='h',cex = 2, xaxs = "i", yaxs = "i", xlim = c(0,chrom$genome.length), ylim = ylim,

main = title, cex.main = 2, ylab = "gistic score", xlab = NA,

cex.lab = 2, col = adjustcolor("darkred", alpha.f = .8), xaxt = "n", lwd = 2, las=1) # las=1 rotating axis labels in R

lines(scores.del$Start.geno, scores.del$G.score, type='h', lwd = 2, col = adjustcolor("midnightblue", alpha.f = .8))

ink <- chrom$chromosomes$chrN %in% chrID

yrange = abs(diff(ylim))

m.pos <- c(ylim[1]+0.05,ylim[2]-0.05)

m.mod <- -(chrom$chromosomes$chrN[ink] %% 2) +2

try(text(x = chrom$chromosomes$middle.chr.geno[ink], y = m.pos[m.mod], labels = chrom$chromosomes$chrom[ink], cex = 1))

abline(h = 0.0, col = 1, lwd = 1, lty = 3)

abline(v = c(0,chrom$chromosomes$chr.length.sum), col = 1, lty = 3, lwd = 1)

col1 <- adjustcolor("darkred", alpha.f = .8)

col2 <- adjustcolor("midnightblue", alpha.f = .8)

# The position of the legend can be specified also using the following keywords : "bottomright", "bottom", "bottomleft", "left", "topleft", "top", "topright", "right" and "center".

legend("topleft", c("gain","loss"), cex=0.6, bty="n", fill=c(col1,col2))

### PTC CS2 ###

scores <- read.table(file.path(res.path,"GISTIC/440602/ptc_immclust_cs2.scores.gistic"), sep="\t",header=T,stringsAsFactors = F)

# Important step for accurate length to match back to continual chrom loci

scores[scores$Chromosome==23,"Chromosome"]="X"

scores[scores$Chromosome==24,"Chromosome"]="Y"

chrID <- unname(unlist(chrom$chrom2chr[as.character(paste0("chr",scores$Chromosome))]))

scores$Start.geno <- scores$Start + chrom$chromosomes$chr.length.cumsum[chrID]

scores$End.geno <- scores$End + chrom$chromosomes$chr.length.cumsum[chrID]

# Prepare input data for ploting

scores.amp <- scores[scores$Type=="Amp",]

scores.amp$G.score <- scores.amp$G.score * 1

scores.del <- scores[scores$Type=="Del",]

scores.del$G.score <- scores.del$G.score * -1

scores <- rbind.data.frame(scores.amp,scores.del)

# seg.col = list(gain = "red", outscale.gain = "darkred", loss = "blue", outscale.red = "midnightblue")

ylim <- c(min(scores$G.score)-0.1,max(scores$G.score)+0.1)

title="CN gistic score of ImmClust CS2 (n = 116)"

plot(scores.amp$Start.geno, scores.amp$G.score,

pch = ".", type='h',cex = 2, xaxs = "i", yaxs = "i", xlim = c(0,chrom$genome.length), ylim = ylim,

main = title, cex.main = 2, ylab = "gistic score", xlab = NA,

cex.lab = 2, col = adjustcolor("darkred", alpha.f = .8), xaxt = "n", lwd = 2, las=1) # las=1 rotating axis labels in R

lines(scores.del$Start.geno, scores.del$G.score, type='h', lwd = 2, col = adjustcolor("midnightblue", alpha.f = .8))

ink <- chrom$chromosomes$chrN %in% chrID

yrange = abs(diff(ylim))

m.pos <- c(ylim[1]+0.05,ylim[2]-0.05)

m.mod <- -(chrom$chromosomes$chrN[ink] %% 2) +2

try(text(x = chrom$chromosomes$middle.chr.geno[ink], y = m.pos[m.mod], labels = chrom$chromosomes$chrom[ink], cex = 1))

abline(h = 0.0, col = 1, lwd = 1, lty = 3)

abline(v = c(0,chrom$chromosomes$chr.length.sum), col = 1, lty = 3, lwd = 1)

col1 <- adjustcolor("darkred", alpha.f = .8)

col2 <- adjustcolor("midnightblue", alpha.f = .8)

# The position of the legend can be specified also using the following keywords : "bottomright", "bottom", "bottomleft", "left", "topleft", "top", "topright", "right" and "center".

legend("topleft", c("gain","loss"), cex=0.6, bty="n", fill=c(col1,col2))

### PTC CS3 ###

scores <- read.table(file.path(res.path,"GISTIC/440603/ptc_immclust_cs3.scores.gistic"), sep="\t",header=T,stringsAsFactors = F)

# Important step for accurate length to match back to continual chrom loci

scores[scores$Chromosome==23,"Chromosome"]="X"

scores[scores$Chromosome==24,"Chromosome"]="Y"

chrID <- unname(unlist(chrom$chrom2chr[as.character(paste0("chr",scores$Chromosome))]))

scores$Start.geno <- scores$Start + chrom$chromosomes$chr.length.cumsum[chrID]

scores$End.geno <- scores$End + chrom$chromosomes$chr.length.cumsum[chrID]

# Prepare input data for ploting

scores.amp <- scores[scores$Type=="Amp",]

scores.amp$G.score <- scores.amp$G.score * 1

scores.del <- scores[scores$Type=="Del",]

scores.del$G.score <- scores.del$G.score * -1

scores <- rbind.data.frame(scores.amp,scores.del)

# seg.col = list(gain = "red", outscale.gain = "darkred", loss = "blue", outscale.red = "midnightblue")

ylim <- c(min(scores$G.score)-0.1,max(scores$G.score)+0.1)

title="CN gistic score of ImmClust CS3 (n = 251)"

plot(scores.amp$Start.geno, scores.amp$G.score,

pch = ".", type='h',cex = 2, xaxs = "i", yaxs = "i", xlim = c(0,chrom$genome.length), ylim = ylim,

main = title, cex.main = 2, ylab = "gistic score", xlab = NA,

cex.lab = 2, col = adjustcolor("darkred", alpha.f = .8), xaxt = "n", lwd = 2, las=1) # las=1 rotating axis labels in R

lines(scores.del$Start.geno, scores.del$G.score, type='h', lwd = 2, col = adjustcolor("midnightblue", alpha.f = .8))

ink <- chrom$chromosomes$chrN %in% chrID

yrange = abs(diff(ylim))

m.pos <- c(ylim[1]+0.05,ylim[2]-0.05)

m.mod <- -(chrom$chromosomes$chrN[ink] %% 2) +2

try(text(x = chrom$chromosomes$middle.chr.geno[ink], y = m.pos[m.mod], labels = chrom$chromosomes$chrom[ink], cex = 1))

abline(h = 0.0, col = 1, lwd = 1, lty = 3)

abline(v = c(0,chrom$chromosomes$chr.length.sum), col = 1, lty = 3, lwd = 1)

col1 <- adjustcolor("darkred", alpha.f = .8)

col2 <- adjustcolor("midnightblue", alpha.f = .8)

# The position of the legend can be specified also using the following keywords : "bottomright", "bottom", "bottomleft", "left", "topleft", "top", "topright", "right" and "center".

legend("topleft", c("gain","loss"), cex=0.6, bty="n", fill=c(col1,col2))

invisible(dev.off())

#-----------------------------------------#

# use MOVICS to characterize two subtypes #

label <- c("Tumor_Sample_Barcode","Hugo_Symbol","NCBI_Build","Chromosome","Start_Position","End_Position","Strand",

"Variant_Classification","Variant_Type","Reference_Allele","Tumor_Seq_Allele1","Tumor_Seq_Allele2","HGVSp_Short")

maf <- read_tsv(file.path(data.path,"data_mutations.txt"), comment = "#")

maf$Tumor_Sample_Barcode <- paste0(maf$Tumor_Sample_Barcode,"A")

comsam <- intersect(tum.sam.expr,maf$Tumor_Sample_Barcode) # 334

maf <- maf[which(maf$Tumor_Sample_Barcode %in% comsam),label]

write.table(maf, file.path(res.path,"thca_maf_modified.txt"),sep = "\t",row.names = F,col.names = T,quote = F)

# Comprehensive Characterization of Cancer Driver Genes and Mutations DOI: 10.1016/j.cell.2018.02.060

thca.sig <- c("AKT1",

"BRAF",

"DNMT3A",

"EIF1AX",

"HRAS",

"KRAS",

"NRAS",

"NUP93",

"PPM1D")

## mutational frequency

ptc.movics.mut <- list("clust.res" = annCol,

"mo.method" = "thca.immclust")

ptc.movics.mut$clust.res$samID <- rownames(ptc.movics.mut$clust.res)

ptc.movics.mut$clust.res$clust <- sapply(ptc.movics.mut$clust.res$ImmClust,

switch,

"CS1" = 1,

"CS2" = 2,

"CS3" = 3)

mut.binary <- matrix(0,nrow = length(unique(maf$Hugo_Symbol)),ncol = length(unique(maf$Tumor_Sample_Barcode)),dimnames = list(unique(maf$Hugo_Symbol),unique(maf$Tumor_Sample_Barcode)))

for (i in colnames(mut.binary)) {

tmp <- maf[which(maf$Tumor_Sample_Barcode == i),]

tmp <- tmp[which(tmp$Variant_Classification %in% c("Frame_Shift_Del", "Frame_Shift_Ins", "Splice_Site", "Translation_Start_Site","Nonsense_Mutation", "Nonstop_Mutation", "In_Frame_Del","In_Frame_Ins", "Missense_Mutation")),]

for (j in tmp$Hugo_Symbol)

mut.binary[j,i] <- 1

}

mut.binary <- as.data.frame(mut.binary)#; rownames(mut.binary) <- toupper(rownames(mut.binary))

mut.binary <- mut.binary[rowSums(mut.binary) > 0,]

ptc.movics.mut$clust.res <- ptc.movics.mut$clust.res[intersect(rownames(ptc.movics.mut$clust.res),colnames(mut.binary)),]

mut.immclust <- compMut(moic.res = ptc.movics.mut,

mut.matrix = mut.binary, # binary somatic mutation matrix

doWord = TRUE, # generate table in .docx format

doPlot = TRUE, # draw OncoPrint

freq.cutoff = 0.01, # keep those genes that mutated in at least 10% of samples

p.cutoff = 0.05, # keep those genes with nominal p value < 0.05 to draw OncoPrint

p.adj.cutoff = 2, # keep those genes with adjusted p value < 0.05 to draw OncoPrint

innerclust = T, # perform clustering within each subtype

annCol = annCol[colnames(mut.binary),colnames(annCol)[c(13,16)]], # same annotation for heatmap

annColors = annColors[c(13,16)], # same annotation color for heatmap

width = 6,

height = 2.5,

fig.name = "oncoprint for siginificant mutations",

tab.name = "independent test between immclust and mutation",

res.path = res.path,

fig.path = fig.path)

## TMB

maf.ptc <- read_tsv(file.path(res.path,"thca_maf_modified.txt"), comment = "#")

tmb.immclust <- compTMB(moic.res = ptc.movics.mut,

maf = maf.ptc,

clust.col = npg[1:3],

rmDup = TRUE, # remove duplicated variants per sample

rmFLAGS = FALSE, # keep FLAGS mutations

exome.size = 38, # estimated exome size

test.method = "nonparametric", # statistical testing method

fig.path = fig.path,

fig.name = "distribution of TMB and TiTv")

segment <- cnv[,c(1:4,6)]

colnames(segment) <- c("sample","chrom","start","end","value")

ptc.movics.cna <- list("clust.res" = annCol,

"mo.method" = "thca.immclust")

ptc.movics.cna$clust.res$samID <- rownames(ptc.movics.cna$clust.res)

ptc.movics.cna$clust.res$clust <- sapply(ptc.movics.cna$clust.res$ImmClust,

switch,

"CS1" = 1,

"CS2" = 2,

"CS3" = 3)

ptc.movics.cna$clust.res <- ptc.movics.cna$clust.res[intersect(rownames(ptc.movics.cna$clust.res),unique(segment$sample)),]

## FGA

fga.immclust <- compFGA(moic.res = ptc.movics.cna,

segment = segment,

clust.col = npg[1:3],

iscopynumber = FALSE, # this is a segmented copy number file

cnathreshold = 0.1, # threshold to determine CNA gain or loss

test.method = "nonparametric", # statistical testing method

fig.path = fig.path,

width = 6,

height = 2.5,

fig.name = "barplot of fga")

## clinical features

tmp <- annCol[,c(1:8,12,13,14,15,16)]

tmp$pStage <- as.character(tmp$pStage)

tmp$tStage <- as.character(tmp$tStage)

tmp$nStage <- as.character(tmp$nStage)

tmp$mStage <- as.character(tmp$mStage)

tmp$Lymphnode <- as.character(tmp$Lymphnode)

tmp$Location <- as.character(tmp$Location)

tmp[tmp == "Missing"] <- NA

clin.immclust <- MOVICS::compClinvar(moic.res = ptc.movics,

includeNA = FALSE,

res.path = res.path,

var2comp = tmp, # data.frame needs to summarize (must has row names of samples)

strata = "ImmClust", # stratifying variable (e.g., Subtype in this example)

factorVars = c("Age","Sex","pStage","tStage","nStage","mStage","Lymphnode","Location","CAPN8_Group","E2F.Clust"), # features that are considered categorical variables

nonnormalVars = c("IES","SES"), # feature(s) that are considered using nonparametric test

exactVars = c("Age","Sex","pStage","tStage","nStage","mStage","Lymphnode","Location","CAPN8_Group","E2F.Clust"), # feature(s) that are considered using exact test

doWord = TRUE, # generate .docx file in local path

tab.name = "summarization of clinical features between cimp in tcga thca")

#----------------------------------------------------------------#

# differential expression analysis and GSEA from two E2F cluster #

pd <- data.frame(Samples = rownames(annCol),

Group = annCol$E2F.Clust,

stringsAsFactors = FALSE)

design <-model.matrix(~ -1 + factor(pd$Group, levels = c("CS1","CS2")))

colnames(design) <- c("CS1","CS2")

gset <- tpm[Mids,pd$Samples]

gset <- gset[rowSums(gset) > 0, ]

fit <- limma::lmFit(gset, design = design);

contrastsMatrix <- limma::makeContrasts(CS1 - CS2, levels = c("CS1", "CS2"))

fit2 <- limma::contrasts.fit(fit, contrasts = contrastsMatrix)

fit2 <- limma::eBayes(fit2, robust = T)

resData <- limma::topTable(fit2, adjust = "fdr", sort.by = "B", number = 100000, confint = T)

resData <- as.data.frame(subset(resData, select=c("logFC","t","B","P.Value","adj.P.Val","CI.L","CI.R")))

resData$id <- rownames(resData)

colnames(resData) <- c("log2fc","t","B","pvalue","padj","CI.L","CI.R","id")

resData$fc <- 2^resData$log2fc

resData <- resData[order(resData$padj),c("id","fc","log2fc","pvalue","padj","CI.L","CI.R")]

write.table(resData,file.path(res.path,"limma results of mrna between E2F cluster in tcga.txt"),sep = "\t",row.names = F,col.names = T,quote = F)

geneList <- resData$log2fc; names(geneList) <- resData$id

geneList <- sort(geneList,decreasing = T)

tmp <- read.gmt(MSIGDB.HALLMARK)

gsea.e2f <- GSEA(geneList = geneList,

TERM2GENE = tmp,

pvalueCutoff = 1,

seed = T,

verbose = F)

gsea.e2f.df <- as.data.frame(gsea.e2f)

write.csv(gsea.e2f.df,file.path(res.path,"gsea hallmark between E2F cluster in tcga thca.csv"),row.names = F,quote = F)

gseaplot2(gsea.e2f, geneSetID = c("HALLMARK_E2F_TARGETS",

"HALLMARK_EPITHELIAL_MESENCHYMAL_TRANSITION",

"HALLMARK_INFLAMMATORY_RESPONSE",

"HALLMARK_INTERFERON_ALPHA_RESPONSE",

"HALLMARK_INTERFERON_GAMMA_RESPONSE"),

pvalue_table = F,color = npg[1:5])

ggsave(filename = file.path(fig.path,"gsea between E2F cluster in tcga thca.pdf"), width = 6,height = 6)

#------------------------#

# DNA replication stress #

HRS.signature <- gmt2list(file.path(comAnn.path,"high_replication_stress.gmt"))

HRS.signature <- sapply(HRS.signature, function(x) setdiff(x,""))

HRS.tcga <- gsva(as.matrix(tpm[,rownames(annCol)]),

HRS.signature,

method = "gsva")

plotdata <- standarize.fun(HRS.tcga[,rownames(annCol)],halfwidth = 1)

rownames(plotdata) <- gsub("REACTOME_","",rownames(plotdata))

rownames(plotdata) <- gsub("HALLMARK_","",rownames(plotdata))

hm1 <- pheatmap(plotdata,

border_color = NA,

show_rownames = TRUE,

show_colnames = F,

#use_raster = FALSE,

cluster_rows = FALSE,

cluster_cols = hcs.thca,

cellheight = 12,

cutree_cols = 2,

cellwidth = 400/ncol(plotdata),

name = "GSVA",

annotation_col = annCol[colnames(plotdata),c("ImmClust","E2F.Clust")],

annotation_colors = annColors[c("ImmClust","E2F.Clust")],

color = viridis(64))

pdf(file = file.path(fig.path, "heatmap of replication stress of E2F cluster to immclust in tcga.pdf"), width = 15,height = 10)

draw(hm1, heatmap_legend_side = "bottom",annotation_legend_side = "bottom")

invisible(dev.off())

# drug sensitivity analysis

gdsc1 <- read.table(file.path(comAnn.path,"GDSC1_fitted_dose_response_25Feb20.txt"),sep = "\t",row.names = NULL,check.names = F,stringsAsFactors = F,header = T)

gdsc2 <- read.table(file.path(comAnn.path,"GDSC2_fitted_dose_response_25Feb20.txt"),sep = "\t",row.names = NULL,check.names = F,stringsAsFactors = F,header = T)

gdsc <- rbind.data.frame(gdsc1, gdsc2); gdsc.bk <- gdsc

gdsc <- gdsc2; gdsc.bk <- gdsc

gdsc$index <- paste(gdsc$CELL_LINE_NAME, gdsc$DRUG_NAME)

gdsc <- gdsc[!duplicated(gdsc$index),c("CELL_LINE_NAME","DRUG_NAME","LN_IC50")]

gdsc.auc <- reshape(gdsc, idvar = "CELL_LINE_NAME", timevar = "DRUG_NAME", direction = "wide")

gdsc.auc$CELL_LINE_NAME <- gsub("-","",gdsc.auc$CELL_LINE_NAME,fixed = T)

gdsc.auc <- gdsc.auc[!duplicated(gdsc.auc$CELL_LINE_NAME),]

rownames(gdsc.auc) <- gdsc.auc$CELL_LINE_NAME

gdsc.auc <- gdsc.auc[,-1]

colnames(gdsc.auc) <- gsub("LN_IC50.","",colnames(gdsc.auc),fixed = T)

load(file.path(comAnn.path,"GDSC_EXPR.RData"))

gdsc.expr <- expr

comccl <- intersect(colnames(gdsc.expr),rownames(gdsc.auc))

gdsc.expr <- gdsc.expr[,comccl]

gdsc.auc <- gdsc.auc[comccl,]

gdsc.auc <- gdsc.auc[,apply(gdsc.auc,2,function(x) sum(is.na(x))) < 0.2*nrow(gdsc.auc)]

gdsc.auc.knn <- as.data.frame(impute.knn(as.matrix(gdsc.auc))$data)

ATR.inhibitor <- unique(gdsc.bk[which(gdsc.bk$PUTATIVE_TARGET == "ATR"),"DRUG_NAME"])

WEE1.inhibitor <- unique(gdsc.bk[which(gdsc.bk$PUTATIVE_TARGET %in% c("WEE1, CHEK1","WEE1, PLK1")),"DRUG_NAME"])

gdsc.auc.knn <- gdsc.auc.knn[,c(ATR.inhibitor,WEE1.inhibitor)]

# tcga

trainExpr <- gdsc.expr

trainPtype <- as.data.frame(gdsc.auc.knn)

comccl <- intersect(rownames(trainPtype),colnames(trainExpr))

trainExpr <- trainExpr[,comccl]

trainPtype <- trainPtype[comccl,]

testExpr <- tpm[,rownames(annCol)]

comgene <- intersect(rownames(trainExpr),rownames(testExpr))

trainExpr <- as.matrix(trainExpr[comgene,])

testExpr <- testExpr[comgene,]

outTab <- NULL

for (i in 1:ncol(trainPtype)) {

display.progress(index = i,totalN = ncol(trainPtype))

d <- colnames(trainPtype)[i]

#tmp <- log2(as.vector(trainPtype[,d]) + 0.00001)

tmp <- as.vector(trainPtype[,d])

ptypeOut <- quiet(calcPhenotype(trainingExprData = as.matrix(trainExpr),

trainingPtype = tmp,

testExprData = as.matrix(testExpr),

powerTransformPhenotype = T,

selection = 1))

#ptypeOut <- 2^ptypeOut - 0.00001

outTab <- rbind.data.frame(outTab,ptypeOut)

}

dimnames(outTab) <- list(colnames(trainPtype),colnames(testExpr))

gdsc.pred.auc.tcga <- as.data.frame(t(outTab))

gdsc.pred.auc.tcga$E2F.Clust <- annCol[rownames(gdsc.pred.auc.tcga),"E2F.Clust"]

wp <- c()

for (i in 1:5) {

tmp1 <- gdsc.pred.auc.tcga[which(gdsc.pred.auc.tcga$E2F.Clust %in% c("CS1")),i]

tmp2 <- gdsc.pred.auc.tcga[which(gdsc.pred.auc.tcga$E2F.Clust %in% c("CS2")),i]

wp <- c(wp,t.test(tmp1,tmp2)$p.value)

}

wp <- as.character(cut(wp,c(0,0.001,0.01,0.05,0.1,1),labels = c("***","**","*",".","")))

names(wp) <- colnames(gdsc.pred.auc.tcga)[1:5]

data_long <- gather(gdsc.pred.auc.tcga, drug, IC50, `VE-822`:`MK-1775`, factor_key=TRUE)

data_long$sig <- wp[data_long$drug]

p3 <- ggplot(data_long, aes(drug, IC50, fill=E2F.Clust)) +

geom_boxplot(aes(col = E2F.Clust),outlier.shape = NA) +

geom_text(aes(drug, y=max(IC50)),

label=data_long$sig,

data=data_long,

inherit.aes=F) +

scale_fill_manual(values = jco[2:1]) +

scale_color_manual(values = jco[2:1]) +

xlab(NULL) + ylab("Estimated IC50") +

theme_bw()+

theme(axis.text.x = element_text(angle = 90, hjust = 1,vjust = 0.5,size = 10, colour = "black"),

legend.position = "bottom",

panel.background = element_blank(),

panel.grid = element_blank(),

legend.title = element_blank())

dat <- ggplot_build(p3)$data[[1]]

p3 <- p3 + geom_segment(data=dat, aes(x=xmin, xend=xmax, y=middle, yend=middle), color="white", inherit.aes = F)

p3

ggsave(file.path(fig.path,"ATR and WEE1 inhibitor in E2F cluster in tcga cohort.pdf"), width = 2,height = 4)

#--------------------------#

# validation in GEO cohort #

## GPL570

mcp.gpl570 <- MCPcounter.estimate(as.matrix(geo.gpl570.expr.combat),

featuresType = "HUGO_symbols",

genes = read.table(file.path(comAnn.path,"genes.txt"),sep = "\t",stringsAsFactors = F,header = T,colClasses = "character",check.names = F),

probesets = read.table(file.path(comAnn.path,"probesets.txt"),sep = "\t",stringsAsFactors = F,header = F,colClasses = "character",check.names = F))

indata <- geo.gpl570.expr.combat

write.table(indata,file = file.path(res.path,"GEO_GPL570_hugo.txt"),sep = "\t",row.names = T,col.names = NA,quote = F)

filterCommonGenes(input.f=file.path(res.path, "GEO_GPL570_hugo.txt") , output.f=file.path(res.path,"GEO_GPL570_hugo_ESTIMATE.txt"), id="GeneSymbol")

estimateScore(file.path(res.path,"GEO_GPL570_hugo_ESTIMATE.txt"), file.path(res.path,"GEO_GPL570_hugo_estimate_score.txt"), platform="affymetrix")

est.geo.gpl570 <- read.table(file = file.path(res.path,"GEO_GPL570_hugo_estimate_score.txt"),header = T,row.names = NULL,check.names = F,stringsAsFactors = F,sep = "\t")

rownames(est.geo.gpl570) <- est.geo.gpl570[,2]; colnames(est.geo.gpl570) <- est.geo.gpl570[1,]; est.geo.gpl570 <- est.geo.gpl570[-1,c(-1,-2)];

est.geo.gpl570 <- sapply(est.geo.gpl570, as.numeric); rownames(est.geo.gpl570) <- c("StromalScore","ImmuneScore","ESTIMATEScore","TumorPurity"); est.geo.gpl570.backup = as.data.frame(est.geo.gpl570); colnames(est.geo.gpl570.backup) <- colnames(indata)

est.geo.gpl570 <- annTrackScale(indata = est.geo.gpl570, halfwidth = 2, poolsd = F); est.geo.gpl570 <- as.data.frame(t(est.geo.gpl570))

rownames(est.geo.gpl570) <- colnames(geo.gpl570.expr.combat)

HRS.geo.gpl570 <- gsva(as.matrix(geo.gpl570.expr.combat),

HRS.signature,

method = "gsva")

annCol.geo.gpl570 <- geo.gpl570.sinfo

annCol.geo.gpl570$IES <- est.geo.gpl570[rownames(annCol.geo.gpl570),"ImmuneScore"]

annCol.geo.gpl570$SES <- est.geo.gpl570[rownames(annCol.geo.gpl570),"StromalScore"]

annCol.geo.gpl570$CAPN8 <- as.numeric(geo.gpl570.expr.combat["CAPN8",rownames(annCol.geo.gpl570)])

annCol.geo.gpl570$CAPN8_Group <- ifelse(annCol.geo.gpl570$CAPN8 > median(annCol.geo.gpl570$CAPN8),"CAPN8.H","CAPN8.L")

tmp <- annCol.geo.gpl570$CAPN8; names(tmp) <- rownames(annCol.geo.gpl570)

annCol.geo.gpl570$CAPN8 <- annTrackScale(tmp, halfwidth = 2)

annColors.geo.gpl570 <- list()

annColors.geo.gpl570[["Batch"]] <- c("GSE3467" = npg[1],"GSE3678" = npg[2],"GSE33630" = npg[3],"GSE60542" = npg[4])

annColors.geo.gpl570[["IES"]] <- annColors.geo.gpl570[["SES"]] <- bluered(64)

annColors.geo.gpl570[["E2F.Clust"]] <- c("CS1" = jco[2], "CS2" = jco[1])

annColors.geo.gpl570[["ImmClust"]] <- c("CS1" = npg[1], "CS2" = npg[2],"CS3" = npg[3])

annColors.geo.gpl570[["CAPN8"]] <- annColors$CAPN8

annColors.geo.gpl570[["CAPN8_Group"]] <- annColors$CAPN8_Group

indata1 <- t(scale(t(geo.gpl570.expr.combat[intersect(rownames(geo.gpl570.expr.combat),e2f.targets),rownames(annCol.geo.gpl570)])))

indata2 <- t(scale(t(rbind.data.frame(geo.gpl570.expr.combat[imm.targets,rownames(annCol.geo.gpl570)],mcp.gpl570[,rownames(annCol.geo.gpl570)]))))

indata <- rbind.data.frame(indata1,indata2)

hcs.geo.gpl570 <- hclust(distanceMatrix(as.matrix(indata), "euclidean"), "ward.D")

hcs.geo.gpl570 <- click_rotate(as.dendrogram(hcs.geo.gpl570))

hcs.geo.gpl570 <- click_rotate(as.dendrogram(hcs.geo.gpl570))

hcs.geo.gpl570 <- as.hclust(hcs.geo.gpl570)

hcg <- hclust(distanceMatrix(as.matrix(t(indata1)), "pearson"), "ward.D")

group <- cutree(hcs.geo.gpl570, k = 2)

group <- paste0("CS",group); names(group) <- colnames(indata)

annCol.geo.gpl570$E2F.Clust <- group

group2 <- cutree(hcs.geo.gpl570, k = 3)

group2 <- paste0("CS",group2); names(group2) <- colnames(indata)

annCol.geo.gpl570$ImmClust <- group2

annCol.geo.gpl570[which(annCol.geo.gpl570$ImmClust == "CS3"),"ImmClust"] <- "CS1 "

annCol.geo.gpl570[which(annCol.geo.gpl570$ImmClust == "CS1"),"ImmClust"] <- "CS2 "

annCol.geo.gpl570[which(annCol.geo.gpl570$ImmClust == "CS2"),"ImmClust"] <- "CS3 "

annCol.geo.gpl570$ImmClust <- gsub(" ","",annCol.geo.gpl570$ImmClust)

plotdata <- standarize.fun(indata1, halfwidth = 2)

hm1 <- pheatmap(plotdata,

cluster_rows = hcg,

cluster_cols = hcs.geo.gpl570,

use_raster = F,

show_colnames = F,

show_rownames = F,

color = NMF:::ccRamp(heatmap.BlBkRd,64),

border_color = NA,

name = "Expr.",

cellwidth = 400/ncol(plotdata),

cellheight = 300/nrow(plotdata),

annotation_col = annCol.geo.gpl570[colnames(plotdata),c("E2F.Clust","ImmClust","Batch","CAPN8","CAPN8_Group","IES","SES")],

annotation_colors = annColors.geo.gpl570[c("E2F.Clust","ImmClust","Batch","CAPN8","CAPN8_Group","IES","SES")])

plotdata <- standarize.fun(geo.gpl570.expr.combat[imm.targets,rownames(annCol.geo.gpl570)],halfwidth = 2)

hm2 <- pheatmap(plotdata,

border_color = NA,

show_rownames = TRUE,

show_colnames = F,

#use_raster = FALSE,

cluster_rows = FALSE,

cluster_cols = hcs.geo.gpl570,

cellheight = 12,

cutree_cols = 2,

cellwidth = 400/ncol(plotdata),

name = "Immunotherapy targets",

color = colorpanel(64,low=blue,mid = "black",high=gold))

plotdata <- standarize.fun(mcp.gpl570[,rownames(annCol.geo.gpl570)],halfwidth = 2)

hm3 <- pheatmap(plotdata,

border_color = NA,

show_rownames = TRUE,

show_colnames = F,

#use_raster = FALSE,

cluster_rows = FALSE,

cluster_cols = hcs.geo.gpl570,

cellheight = 12,

cutree_cols = 2,

cellwidth = 400/ncol(plotdata),

name = "MCPcounter",

color = greenred(64))

plotdata <- standarize.fun(HRS.geo.gpl570[,rownames(annCol.geo.gpl570)],halfwidth = 1)

rownames(plotdata) <- gsub("REACTOME_","",rownames(plotdata))

rownames(plotdata) <- gsub("HALLMARK_","",rownames(plotdata))

hm4 <- pheatmap(plotdata,

border_color = NA,

show_rownames = TRUE,

show_colnames = F,

#use_raster = FALSE,

cluster_rows = FALSE,

cluster_cols = hcs.geo.gpl570,

cellheight = 12,

cutree_cols = 2,

cellwidth = 400/ncol(plotdata),

name = "GSVA",

color = viridis(64))

pdf(file.path(fig.path,"unsupervised heatmap of e2f targets genes in geo gpl570 thca.pdf"), width = 15,height = 15)

draw(hm1 %v% hm2 %v% hm3 %v% hm4, heatmap_legend_side = "bottom",annotation_legend_side = "bottom")

invisible(dev.off())

trainExpr <- gdsc.expr

trainPtype <- as.data.frame(gdsc.auc.knn)

comccl <- intersect(rownames(trainPtype),colnames(trainExpr))

trainExpr <- trainExpr[,comccl]

trainPtype <- trainPtype[comccl,]

testExpr <- geo.gpl570.expr.combat[,rownames(annCol.geo.gpl570)]

comgene <- intersect(rownames(trainExpr),rownames(testExpr))

trainExpr <- as.matrix(trainExpr[comgene,])

testExpr <- testExpr[comgene,]

outTab <- NULL

for (i in 1:ncol(trainPtype)) {

display.progress(index = i,totalN = ncol(trainPtype))

d <- colnames(trainPtype)[i]

#tmp <- log2(as.vector(trainPtype[,d]) + 0.00001)

tmp <- as.vector(trainPtype[,d])

ptypeOut <- quiet(calcPhenotype(trainingExprData = as.matrix(trainExpr),

trainingPtype = tmp,

testExprData = as.matrix(testExpr),

powerTransformPhenotype = T,

selection = 1))

#ptypeOut <- 2^ptypeOut - 0.00001

outTab <- rbind.data.frame(outTab,ptypeOut)

}

dimnames(outTab) <- list(colnames(trainPtype),colnames(testExpr))

gdsc.pred.auc.gpl570 <- as.data.frame(t(outTab))

gdsc.pred.auc.gpl570$E2F.Clust <- annCol.geo.gpl570[rownames(gdsc.pred.auc.gpl570),"E2F.Clust"]

wp <- c()

for (i in 1:5) {

tmp1 <- gdsc.pred.auc.gpl570[which(gdsc.pred.auc.gpl570$E2F.Clust %in% c("CS1")),i]

tmp2 <- gdsc.pred.auc.gpl570[which(gdsc.pred.auc.gpl570$E2F.Clust %in% c("CS2")),i]

wp <- c(wp,t.test(tmp1,tmp2)$p.value)

}

wp <- as.character(cut(wp,c(0,0.001,0.01,0.05,0.1,1),labels = c("***","**","*",".","")))

names(wp) <- colnames(gdsc.pred.auc.gpl570)[1:5]

data_long <- gather(gdsc.pred.auc.gpl570, drug, IC50, `VE-822`:`MK-1775`, factor_key=TRUE)

data_long$sig <- wp[data_long$drug]

p3 <- ggplot(data_long, aes(drug, IC50, fill=E2F.Clust)) +

geom_boxplot(aes(col = E2F.Clust),outlier.shape = NA) +

geom_text(aes(drug, y=max(IC50)),

label=data_long$sig,

data=data_long,

inherit.aes=F) +

scale_fill_manual(values = jco[2:1]) +

scale_color_manual(values = jco[2:1]) +

xlab(NULL) + ylab("Estimated IC50") +

theme_bw()+

theme(axis.text.x = element_text(angle = 90, hjust = 1,vjust = 0.5,size = 10, colour = "black"),

legend.position = "bottom",

panel.background = element_blank(),

panel.grid = element_blank(),

legend.title = element_blank())

dat <- ggplot_build(p3)$data[[1]]

p3 <- p3 + geom_segment(data=dat, aes(x=xmin, xend=xmax, y=middle, yend=middle), color="white", inherit.aes = F)

p3

ggsave(file.path(fig.path,"ATR and WEE1 inhibitor in E2F cluster in gpl570 cohort.pdf"), width = 2,height = 4)

## GPL96

mcp.gpl96 <- MCPcounter.estimate(as.matrix(geo.gpl96.expr),

featuresType = "HUGO_symbols",

genes = read.table(file.path(comAnn.path,"genes.txt"),sep = "\t",stringsAsFactors = F,header = T,colClasses = "character",check.names = F),

probesets = read.table(file.path(comAnn.path,"probesets.txt"),sep = "\t",stringsAsFactors = F,header = F,colClasses = "character",check.names = F))

indata <- geo.gpl96.expr

write.table(indata,file = file.path(res.path,"GEO_GPL96_hugo.txt"),sep = "\t",row.names = T,col.names = NA,quote = F)

filterCommonGenes(input.f=file.path(res.path, "GEO_GPL96_hugo.txt") , output.f=file.path(res.path,"GEO_GPL96_hugo_ESTIMATE.txt"), id="GeneSymbol")

estimateScore(file.path(res.path,"GEO_GPL96_hugo_ESTIMATE.txt"), file.path(res.path,"GEO_GPL96_hugo_estimate_score.txt"), platform="affymetrix")

est.geo.gpl96 <- read.table(file = file.path(res.path,"GEO_GPL96_hugo_estimate_score.txt"),header = T,row.names = NULL,check.names = F,stringsAsFactors = F,sep = "\t")

rownames(est.geo.gpl96) <- est.geo.gpl96[,2]; colnames(est.geo.gpl96) <- est.geo.gpl96[1,]; est.geo.gpl96 <- est.geo.gpl96[-1,c(-1,-2)];

est.geo.gpl96 <- sapply(est.geo.gpl96, as.numeric); rownames(est.geo.gpl96) <- c("StromalScore","ImmuneScore","ESTIMATEScore","TumorPurity"); est.geo.gpl96.backup = as.data.frame(est.geo.gpl96); colnames(est.geo.gpl96.backup) <- colnames(indata)

est.geo.gpl96 <- annTrackScale(indata = est.geo.gpl96, halfwidth = 2, poolsd = F); est.geo.gpl96 <- as.data.frame(t(est.geo.gpl96))

rownames(est.geo.gpl96) <- colnames(geo.gpl96.expr)

HRS.geo.gpl96 <- gsva(as.matrix(geo.gpl96.expr),

HRS.signature,

method = "gsva")

annCol.geo.gpl96 <- geo.gpl96.sinfo

annCol.geo.gpl96$IES <- est.geo.gpl96[rownames(annCol.geo.gpl96),"ImmuneScore"]

annCol.geo.gpl96$SES <- est.geo.gpl96[rownames(annCol.geo.gpl96),"StromalScore"]

annColors.geo.gpl96 <- list()

annColors.geo.gpl96[["IES"]] <- annColors.geo.gpl96[["SES"]] <- bluered(64)

annColors.geo.gpl96[["E2F.Clust"]] <- c("CS1" = jco[1], "CS2" = jco[2])

annColors.geo.gpl96[["ImmClust"]] <- c("CS1" = npg[1], "CS2" = npg[2],"CS3" = npg[3])

indata1 <- t(scale(t(geo.gpl96.expr[intersect(rownames(geo.gpl96.expr),e2f.targets),rownames(annCol.geo.gpl96)])))

indata2 <- t(scale(t(rbind.data.frame(geo.gpl96.expr[intersect(imm.targets,rownames(geo.gpl96.expr)),rownames(annCol.geo.gpl96)],mcp.gpl96[,rownames(annCol.geo.gpl96)]))))

indata <- rbind.data.frame(indata1,indata2)

hcs.geo.gpl96 <- hclust(distanceMatrix(as.matrix(indata), "euclidean"), "ward.D")

hcs.geo.gpl96 <- click_rotate(as.dendrogram(hcs.geo.gpl96))

hcs.geo.gpl96 <- as.hclust(hcs.geo.gpl96)

hcg <- hclust(distanceMatrix(as.matrix(t(indata1)), "pearson"), "ward.D")

group <- cutree(hcs.geo.gpl96, k = 2)

group <- paste0("CS",group); names(group) <- colnames(indata)

annCol.geo.gpl96$E2F.Clust <- group

annCol.geo.gpl96[which(annCol.geo.gpl96$E2F.Clust %in% "CS1"),"E2F.Clust"] <- "CS2 "

annCol.geo.gpl96[which(annCol.geo.gpl96$E2F.Clust %in% "CS2"),"E2F.Clust"] <- "CS1 "

annCol.geo.gpl96$E2F.Clust <- gsub(" ","",annCol.geo.gpl96$E2F.Clust)

group2 <- cutree(hcs.geo.gpl96, k = 5)

group2 <- paste0("CS",group2); names(group2) <- colnames(indata)

annCol.geo.gpl96$ImmClust <- group2

annCol.geo.gpl96[which(annCol.geo.gpl96$ImmClust %in% c("CS1","CS2")),"ImmClust"] <- "CS3 "

annCol.geo.gpl96[which(annCol.geo.gpl96$ImmClust %in% c("CS5","CS3")),"ImmClust"] <- "CS2 "

annCol.geo.gpl96[which(annCol.geo.gpl96$ImmClust == "CS4"),"ImmClust"] <- "CS1 "

annCol.geo.gpl96$ImmClust <- gsub(" ","",annCol.geo.gpl96$ImmClust)

plotdata <- standarize.fun(indata1, halfwidth = 2)

hm1 <- pheatmap(plotdata,

cluster_rows = hcg,

cluster_cols = hcs.geo.gpl96,

use_raster = F,

show_colnames = F,

show_rownames = F,

color = NMF:::ccRamp(heatmap.BlBkRd,64),

border_color = NA,

name = "Expr.",

cellwidth = 400/ncol(plotdata),

cellheight = 300/nrow(plotdata),

annotation_col = annCol.geo.gpl96[colnames(plotdata),c("E2F.Clust","ImmClust","IES","SES")],

annotation_colors = annColors.geo.gpl96[c("E2F.Clust","ImmClust","IES","SES")])

plotdata <- standarize.fun(geo.gpl96.expr[intersect(imm.targets,rownames(geo.gpl96.expr)),rownames(annCol.geo.gpl96)],halfwidth = 2)

hm2 <- pheatmap(plotdata,

border_color = NA,

show_rownames = TRUE,

show_colnames = F,

#use_raster = FALSE,

cluster_rows = FALSE,

cluster_cols = hcs.geo.gpl96,

cellheight = 12,

cutree_cols = 2,

cellwidth = 400/ncol(plotdata),

name = "Immunotherapy targets",

color = colorpanel(64,low=blue,mid = "black",high=gold))

plotdata <- standarize.fun(mcp.gpl96[,rownames(annCol.geo.gpl96)],halfwidth = 2)

hm3 <- pheatmap(plotdata,

border_color = NA,

show_rownames = TRUE,

show_colnames = F,

#use_raster = FALSE,

cluster_rows = FALSE,

cluster_cols = hcs.geo.gpl96,

cellheight = 12,

cutree_cols = 2,

cellwidth = 400/ncol(plotdata),

name = "MCPcounter",

color = greenred(64))

plotdata <- standarize.fun(HRS.geo.gpl96[,rownames(annCol.geo.gpl96)],halfwidth = 1)

rownames(plotdata) <- gsub("REACTOME_","",rownames(plotdata))

rownames(plotdata) <- gsub("HALLMARK_","",rownames(plotdata))

hm4 <- pheatmap(plotdata,

border_color = NA,

show_rownames = TRUE,

show_colnames = F,

#use_raster = FALSE,

cluster_rows = FALSE,

cluster_cols = hcs.geo.gpl96,

cellheight = 12,

cutree_cols = 2,

cellwidth = 400/ncol(plotdata),

name = "GSVA",

color = viridis(64))

pdf(file.path(fig.path,"unsupervised heatmap of e2f targets genes in geo gpl96 thca.pdf"), width = 15,height = 15)

draw(hm1 %v% hm2 %v% hm3 %v% hm4, heatmap_legend_side = "bottom",annotation_legend_side = "bottom")

invisible(dev.off())

trainExpr <- gdsc.expr

trainPtype <- as.data.frame(gdsc.auc.knn)

comccl <- intersect(rownames(trainPtype),colnames(trainExpr))

trainExpr <- trainExpr[,comccl]

trainPtype <- trainPtype[comccl,]

testExpr <- geo.gpl96.expr[,rownames(annCol.geo.gpl96)]

comgene <- intersect(rownames(trainExpr),rownames(testExpr))

trainExpr <- as.matrix(trainExpr[comgene,])

testExpr <- testExpr[comgene,]

outTab <- NULL

for (i in 1:ncol(trainPtype)) {

display.progress(index = i,totalN = ncol(trainPtype))

d <- colnames(trainPtype)[i]

#tmp <- log2(as.vector(trainPtype[,d]) + 0.00001)

tmp <- as.vector(trainPtype[,d])

ptypeOut <- quiet(calcPhenotype(trainingExprData = as.matrix(trainExpr),

trainingPtype = tmp,

testExprData = as.matrix(testExpr),

powerTransformPhenotype = T,

selection = 1))

#ptypeOut <- 2^ptypeOut - 0.00001

outTab <- rbind.data.frame(outTab,ptypeOut)

}

dimnames(outTab) <- list(colnames(trainPtype),colnames(testExpr))

gdsc.pred.auc.gpl96 <- as.data.frame(t(outTab))

gdsc.pred.auc.gpl96$E2F.Clust <- annCol.geo.gpl96[rownames(gdsc.pred.auc.gpl96),"E2F.Clust"]

wp <- c()

for (i in 1:5) {

tmp1 <- gdsc.pred.auc.gpl96[which(gdsc.pred.auc.gpl96$E2F.Clust %in% c("CS1")),i]

tmp2 <- gdsc.pred.auc.gpl96[which(gdsc.pred.auc.gpl96$E2F.Clust %in% c("CS2")),i]

wp <- c(wp,t.test(tmp1,tmp2)$p.value)

}

wp <- as.character(cut(wp,c(0,0.001,0.01,0.05,0.1,1),labels = c("***","**","*",".","")))

names(wp) <- colnames(gdsc.pred.auc.gpl96)[1:5]

data_long <- gather(gdsc.pred.auc.gpl96, drug, IC50, `VE-822`:`MK-1775`, factor_key=TRUE)

data_long$sig <- wp[data_long$drug]

p3 <- ggplot(data_long, aes(drug, IC50, fill=E2F.Clust)) +

geom_boxplot(aes(col = E2F.Clust),outlier.shape = NA) +

geom_text(aes(drug, y=max(IC50)),

label=data_long$sig,

data=data_long,

inherit.aes=F) +

scale_fill_manual(values = jco[2:1]) +

scale_color_manual(values = jco[2:1]) +

xlab(NULL) + ylab("Estimated IC50") +

theme_bw()+

theme(axis.text.x = element_text(angle = 90, hjust = 1,vjust = 0.5,size = 10, colour = "black"),

legend.position = "bottom",

panel.background = element_blank(),

panel.grid = element_blank(),

legend.title = element_blank())

dat <- ggplot_build(p3)$data[[1]]

p3 <- p3 + geom_segment(data=dat, aes(x=xmin, xend=xmax, y=middle, yend=middle), color="white", inherit.aes = F)

p3

ggsave(file.path(fig.path,"ATR and WEE1 inhibitor in E2F cluster in gpl96 cohort.pdf"), width = 2,height = 4)

# submap analysis

# create submap format for skin melanoma

skcm.immunotherapy.logNC <- read.table(file.path(data.path,"skcm.immunotherapy.47samples.log2CountsNorm.txt"),sep = "\t",row.names = 1,header = T,check.names = F,stringsAsFactors = F) #原文提供的log2转化的标准化count值

rownames(skcm.immunotherapy.logNC) <- toupper(rownames(skcm.immunotherapy.logNC))

skcm.immunotherapy.info <- read.table(file.path(data.path,"skcm.immunotherapy.47sampleInfo.txt"),sep = "\t",row.names = 1,header = T,check.names = F,stringsAsFactors = F)

skcm.immunotherapy.info <- skcm.immunotherapy.info[order(skcm.immunotherapy.info$label),]

skcm.immunotherapy.info$rank <- rep(c(1,2,3,4),times=as.character(table(skcm.immunotherapy.info$label))) #1: CTLA4_noR 2: CTLA4_R 3:PD1_noR 4:PD1_R

# create submap format for tcga

GENELIST <- intersect(rownames(geo.gpl570.expr.combat),rownames(skcm.immunotherapy.logNC))

sam_info <- skcm.immunotherapy.info

in_gct <- skcm.immunotherapy.logNC[GENELIST,rownames(skcm.immunotherapy.info)]

# generate file

gct_file <- file.path(res.path,"skcm2.immunotherapy.for.SubMap.gct")

cls_file <- file.path(res.path,"skcm2.immunotherapy.for.SubMap.cls")

generateInputFileForSubMap(in_gct = in_gct, gct_file = gct_file, cls_file = cls_file, sam_info = sam_info, type_name = "rank")

# extract subtypes

sample.C1 <- rownames(annCol.geo.gpl570[annCol.geo.gpl570$ImmClust == "CS1",])

sample.C2 <- rownames(annCol.geo.gpl570[annCol.geo.gpl570$ImmClust == "CS2",])

sample.C3 <- rownames(annCol.geo.gpl570[annCol.geo.gpl570$ImmClust == "CS3",])

sam_info <- data.frame("ImmClust"=c(sample.C1,sample.C2,sample.C3),row.names = c(sample.C1,sample.C2,sample.C3))

sam_info$rank <- rep(c(1,2,3),times=c(length(sample.C1),length(sample.C2),length(sample.C3)))

# generate file

gct_file <- file.path(res.path,"GPL570.ImmClust.for.SubMap.gct")

cls_file <- file.path(res.path,"GPL570.ImmClust.for.SubMap.cls")

in_gct <- geo.gpl570.expr.combat[GENELIST,rownames(sam_info)]

generateInputFileForSubMap(in_gct = in_gct, gct_file = gct_file, cls_file = cls_file, sam_info = sam_info, type_name = "rank")

# create submap format for skin melanoma

skcm.immunotherapy.logNC <- read.table(file.path(data.path,"skcm.immunotherapy.47samples.log2CountsNorm.txt"),sep = "\t",row.names = 1,header = T,check.names = F,stringsAsFactors = F) #原文提供的log2转化的标准化count值

rownames(skcm.immunotherapy.logNC) <- toupper(rownames(skcm.immunotherapy.logNC))

skcm.immunotherapy.info <- read.table(file.path(data.path,"skcm.immunotherapy.47sampleInfo.txt"),sep = "\t",row.names = 1,header = T,check.names = F,stringsAsFactors = F)

skcm.immunotherapy.info <- skcm.immunotherapy.info[order(skcm.immunotherapy.info$label),]

skcm.immunotherapy.info$rank <- rep(c(1,2,3,4),times=as.character(table(skcm.immunotherapy.info$label))) #1: CTLA4_noR 2: CTLA4_R 3:PD1_noR 4:PD1_R

# create submap format for tcga

GENELIST <- intersect(rownames(geo.gpl96.expr),rownames(skcm.immunotherapy.logNC))

sam_info <- skcm.immunotherapy.info

in_gct <- skcm.immunotherapy.logNC[GENELIST,rownames(skcm.immunotherapy.info)]

# generate file

gct_file <- file.path(res.path,"skcm3.immunotherapy.for.SubMap.gct")

cls_file <- file.path(res.path,"skcm3.immunotherapy.for.SubMap.cls")

generateInputFileForSubMap(in_gct = in_gct, gct_file = gct_file, cls_file = cls_file, sam_info = sam_info, type_name = "rank")

# extract subtypes

sample.C1 <- rownames(annCol.geo.gpl96[annCol.geo.gpl96$ImmClust == "CS1",])

sample.C2 <- rownames(annCol.geo.gpl96[annCol.geo.gpl96$ImmClust == "CS2",])

sample.C3 <- rownames(annCol.geo.gpl96[annCol.geo.gpl96$ImmClust == "CS3",])

sam_info <- data.frame("ImmClust"=c(sample.C1,sample.C2,sample.C3),row.names = c(sample.C1,sample.C2,sample.C3))

sam_info$rank <- rep(c(1,2,3),times=c(length(sample.C1),length(sample.C2),length(sample.C3)))

# generate file

gct_file <- file.path(res.path,"GPL96.ImmClust.for.SubMap.gct")

cls_file <- file.path(res.path,"GPL96.ImmClust.for.SubMap.cls")

in_gct <- geo.gpl96.expr[GENELIST,rownames(sam_info)]

generateInputFileForSubMap(in_gct = in_gct, gct_file = gct_file, cls_file = cls_file, sam_info = sam_info, type_name = "rank")

tmp <- matrix(c(0.849,0.150,0.838,0.001, # nominal p value

0.037,0.096,0.133,0.877,

0.769,0.808,0.541,0.771,

1,1,1,0.012, # Bonferroni adjusted p value

0.444,1,1,1,

1,1,1,1,

0.926,0.360,1,0.012, # FDR adjusted p value

0.222,0.384,0.399,0.877,

1,1,1,1),

nrow = 9,byrow = T,

dimnames = list(c("CS1","CS2","CS3","CS1 ","CS2 ","CS3 ","CS1 ","CS2 ","CS3 "),c("CTAL4-noR","CTLA4-R","PD1-noR","PD1-R")))

pdf(file.path(fig.path,"submap heatmap of predicted response to immunotherapy in tcga thca.pdf"),width = 5,height = 6)

pheatmap(tmp,

border_color = "white",

number_format = "%.3f",

fontsize_number = 9,

cellwidth = 30, cellheight = 30,

cluster_rows = F,cluster_cols = F,

color = rev(NMF:::ccRamp(c("#E6EAF7","#B6D1E8","#498EB9","#204F8D"),64)),

display_numbers = T,

number_color = "black",

name = "Statitic",

annotation_row = data.frame(pvalue=c("Nominal p value","Nominal p value","Nominal p value","Bonferroni adjusted","Bonferroni adjusted","Bonferroni adjusted","FDR adjusted","FDR adjusted","FDR adjusted"),

row.names = rownames(tmp)),

annotation_colors = list(pvalue=c("Nominal p value"="black","Bonferroni adjusted"="grey80","FDR adjusted" = "grey60")))

invisible(dev.off())

tmp <- matrix(c(0.969,0.144,0.990,0.001, # nominal p value

0.738,0.392,0.710,0.041,

0.203,0.375,0.390,0.990,

1,1,1,0.012, # Bonferroni adjusted p value

1,1,1,0.492,

1,1,1,1,

1,0.575,1,0.012, # FDR adjusted p value

0.984,0.671,1,0.246,

0.608,0.899,0.779,1),

nrow = 9,byrow = T,

dimnames = list(c("CS1","CS2","CS3","CS1 ","CS2 ","CS3 ","CS1 ","CS2 ","CS3 "),c("CTAL4-noR","CTLA4-R","PD1-noR","PD1-R")))

pdf(file.path(fig.path,"submap heatmap of predicted response to immunotherapy in gpl570 thca.pdf"),width = 5,height = 6)

pheatmap(tmp,

border_color = "white",

number_format = "%.3f",

fontsize_number = 9,

cellwidth = 30, cellheight = 30,

cluster_rows = F,cluster_cols = F,

color = rev(NMF:::ccRamp(c("#E6EAF7","#B6D1E8","#498EB9","#204F8D"),64)),

display_numbers = T,

number_color = "black",

name = "Statitic",

annotation_row = data.frame(pvalue=c("Nominal p value","Nominal p value","Nominal p value","Bonferroni adjusted","Bonferroni adjusted","Bonferroni adjusted","FDR adjusted","FDR adjusted","FDR adjusted"),

row.names = rownames(tmp)),

annotation_colors = list(pvalue=c("Nominal p value"="black","Bonferroni adjusted"="grey80","FDR adjusted" = "grey60")))

invisible(dev.off())

tmp <- matrix(c(0.862,0.087,0.978,0.016, # nominal p value

0.882,0.093,0.505,0.391,

0.172,0.624,0.487,0.688,

1,1,1,0.012, # Bonferroni adjusted p value

1,0.707,1,1,

1,1,1,1,

1,0.336,0.991,0.012, # FDR adjusted p value

1,0.354,0.388,0.345,

0.427,1,0.908,1),

nrow = 9,byrow = T,

dimnames = list(c("CS1","CS2","CS3","CS1 ","CS2 ","CS3 ","CS1 ","CS2 ","CS3 "),c("CTAL4-noR","CTLA4-R","PD1-noR","PD1-R")))

pdf(file.path(fig.path,"submap heatmap of predicted response to immunotherapy in gpl96 thca.pdf"),width = 5,height = 6)

pheatmap(tmp,

border_color = "white",

number_format = "%.3f",

fontsize_number = 9,

cellwidth = 30, cellheight = 30,

cluster_rows = F,cluster_cols = F,

color = rev(NMF:::ccRamp(c("#E6EAF7","#B6D1E8","#498EB9","#204F8D"),64)),

display_numbers = T,

number_color = "black",

name = "Statitic",

annotation_row = data.frame(pvalue=c("Nominal p value","Nominal p value","Nominal p value","Bonferroni adjusted","Bonferroni adjusted","Bonferroni adjusted","FDR adjusted","FDR adjusted","FDR adjusted"),

row.names = rownames(tmp)),

annotation_colors = list(pvalue=c("Nominal p value"="black","Bonferroni adjusted"="grey80","FDR adjusted" = "grey60")))

invisible(dev.off())

# save image

save.image(file = file.path(workdir,"THCA.CAPN8.RData"))
